# Environmental context and magnitude of disturbance influence trait‐mediated community responses to wastewater in streams

**DOI:** 10.1002/ece3.2165

**Published:** 2016-05-12

**Authors:** Francis J. Burdon, Marta Reyes, Alfredo C. Alder, Adriano Joss, Christoph Ort, Katja Räsänen, Jukka Jokela, Rik I.L. Eggen, Christian Stamm

**Affiliations:** ^1^ EawagSwiss Federal Institute of Aquatic Science and TechnologyDübendorfSwitzerland; ^2^ ETH‐ZurichSwiss Federal Institute of TechnologyZurichSwitzerland

**Keywords:** Land use, macroinvertebrates, multiple stressors, pollution, resistance, sensitivity

## Abstract

Human land uses and population growth represent major global threats to biodiversity and ecosystem services. Understanding how biological communities respond to multiple drivers of human‐induced environmental change is fundamental for conserving ecosystems and remediating degraded habitats. Here, we used a replicated ‘real‐world experiment’ to study the responses of invertebrate communities to wastewater perturbations across a land‐use intensity gradient in 12 Swiss streams. We used different taxonomy and trait‐based community descriptors to establish the most sensitive indicators detecting impacts and to help elucidate potential causal mechanisms of change. First, we predicted that streams in catchments adversely impacted by human land‐uses would be less impaired by wastewater inputs because their invertebrate communities should be dominated by pollution‐tolerant taxa (‘environmental context’). Second, we predicted that the negative effects of wastewater on stream invertebrate communities should be larger in streams that receive proportionally more wastewater (‘magnitude of disturbance’). In support of the ‘environmental context’ hypothesis, we found that change in the Saprobic Index (a trait‐based indicator of tolerance to organic pollution) was associated with upstream community composition; communities in catchments with intensive agricultural land uses (e.g., arable cropping and pasture) were generally more resistant to eutrophication associated with wastewater inputs. We also found support for the ‘magnitude of disturbance’ hypothesis. The SPEAR Index (a trait‐based indicator of sensitivity to pesticides) was more sensitive to the relative input of effluent, suggesting that toxic influences of wastewater scale with dilution. Whilst freshwater pollution continues to be a major environmental problem, our findings highlight that the same anthropogenic pressure (i.e., inputs of wastewater) may induce different ecological responses depending on the environmental context and community metrics used. Thus, remediation strategies aiming to improve stream ecological status (e.g., rehabilitating degraded reaches) need to consider upstream anthropogenic influences and the most appropriate indicators of restoration success.

## Introduction

Human population growth and associated demands on natural resources are changing freshwater ecosystems globally (Vorosmarty et al. 2010). Such changes are often linked to anthropogenic pressures associated with human land uses, including habitat degradation, water abstraction, and diffuse and point‐source pollution (Allan 2004; Friberg 2010). In turn, these pressures can increase environmental stressors, such as inputs of nutrients, pesticides, and fine inorganic sediment that are commonly associated with eutrophication and reductions in pollution‐sensitive taxa (Liess and Ohe 2005; Smith et al. 2006; Burdon et al. 2013). This means that the state of freshwater ecosystems often depends on the magnitude and spatio‐temporal extent of human pressures in the surrounding catchment (Allan 2004; Friberg 2010). Thus, to optimize water management in increasingly common multiple‐stressor scenarios, it is important to understand the relative roles that different pressures take in determining the ecological status of ecosystems (Friberg 2010). Here, we were interested in how catchment land‐uses influence stream invertebrate community responses to point‐source pollution (i.e., inputs of treated wastewater).

The widespread establishment of municipal wastewater treatment plants (WWTPs) has greatly improved surface water quality in developed countries (Vaughan and Ormerod 2012). However, although WWTPs have traditionally been designed to retain and remove organic matter, microbes, and nutrients, these contaminants are often not totally eliminated. In addition, the poor removal of micropollutants (i.e., mixtures of pharmaceuticals, pesticides, etc. in low concentrations) is of major concern because they potentially exert negative influences on aquatic ecosystems (Schwarzenbach et al. 2006; Eggen et al. 2014). Consequently, wastewater can perturb receiving environments through eutrophication and toxicity, thus changing community composition and leading to altered food‐web structure and decreased ecosystem functioning (Singer and Battin 2007; Englert et al. 2013). Typical invertebrate community responses to wastewater inputs include increased densities of pollution‐tolerant oligochaete worms and chironomid dipterans, and decreased diversity and abundances of pollution‐sensitive taxa such as aquatic insects from the orders Ephemeroptera, Trichoptera, and Plecoptera (EPT fauna) (Hynes 1960; Gücker et al. 2006).

Clearly demonstrating the mechanisms driving wastewater impacts (Stalter et al. 2013) reflects a wider problem of ascertaining causal pathways in modified catchments with multiple stressors (Burdon et al. 2013). Disentangling the influence of multiple stressors is a scientific challenge for several reasons. Firstly, influencing factors such as water quality, stream morphology, and temperature are often correlated (Robinson et al. 2014). Secondly, experimental manipulations often lack sufficient spatial and temporal extent to provide meaningful insights into pressures at the catchment level (Cooper et al. 1998). To help resolve these issues, we used a replicated ‘real‐world experiment’ in 12 Swiss streams across a land‐use intensity gradient to investigate stream responses to a single additional pressure not present upstream (i.e., treated wastewater).

Another way to potentially disentangle the effects of multiple stressors is to use trait‐based community descriptors that are specific for certain stressors (Menezes et al. 2010). This approach builds upon the traditional assessments of stream health using occupancy and abundances of identified macroinvertebrate taxa as indicators (Carter et al. 2006). However, using species traits as biomonitoring tools help better link communities to habitat pressures and offer a more mechanistic alternative to traditional taxonomy‐based descriptors (Statzner and Beche 2010). This is important because there is a paucity of knowledge on the environmental contingency of invertebrate responses to different stressors and the best indicators to detect changes (Gücker et al. 2006). We tested responses to wastewater inputs using stream invertebrate community data with both taxonomic and trait‐based approaches.

These challenges reflect the increased demand for knowledge on how freshwater ecosystems respond to various levels of perturbations and at what levels recovery will occur (Friberg 2010). The response of ecological systems to disturbance is an important topic in research regarding ecological stability (Grimm et al. 1992). Ecological stability is described as a multifaceted concept, including properties such as variability, resilience (recovery), and resistance (Donohue et al. 2013). Resistance reflects the ability of communities or populations to remain “essentially unchanged” when subject to disturbance (Grimm and Wissel 1997). The inverse of resistance is sensitivity; sensitive communities should express large structural changes (e.g., reduced diversity) when exposed to a perturbation (Grimm et al. 1992). The observed changes in a community can also be influenced by the different properties of a disturbance, such as its magnitude, frequency, and duration (Pimm 1984).

In this context, changes in response to perturbations can be predicted to be the product of the community's sensitivity and the disturbance (Eq. (1)). This approach corresponds to a (local) sensitivity analysis:

(1) $$\Delta Y_{i}=\frac{\partial Y_{i}}{\partial X}\times \Delta X$$

where *Y* is an ecological metric characterizing the status of an ecosystem. The state of the system at *i* (i.e., a point in time or space) is defined as the ecological status *Y*_*i*_ relative to an existing level of disturbance *X*. Δ*Y*_*i*_ describes the response of the ecosystem to an additional disturbance Δ*X*. By quantifying Δ*X* and Δ*Y*_*i*_, it is possible to test the extent to which metric‐specific sensitivities vary with ecological status. Here, we apply the general approach described in Eq. (1) to assess stream invertebrate community changes in response to an anthropogenic disturbance (i.e., pollution by treated wastewater) across a land‐use intensity gradient.

**Figure 1:**
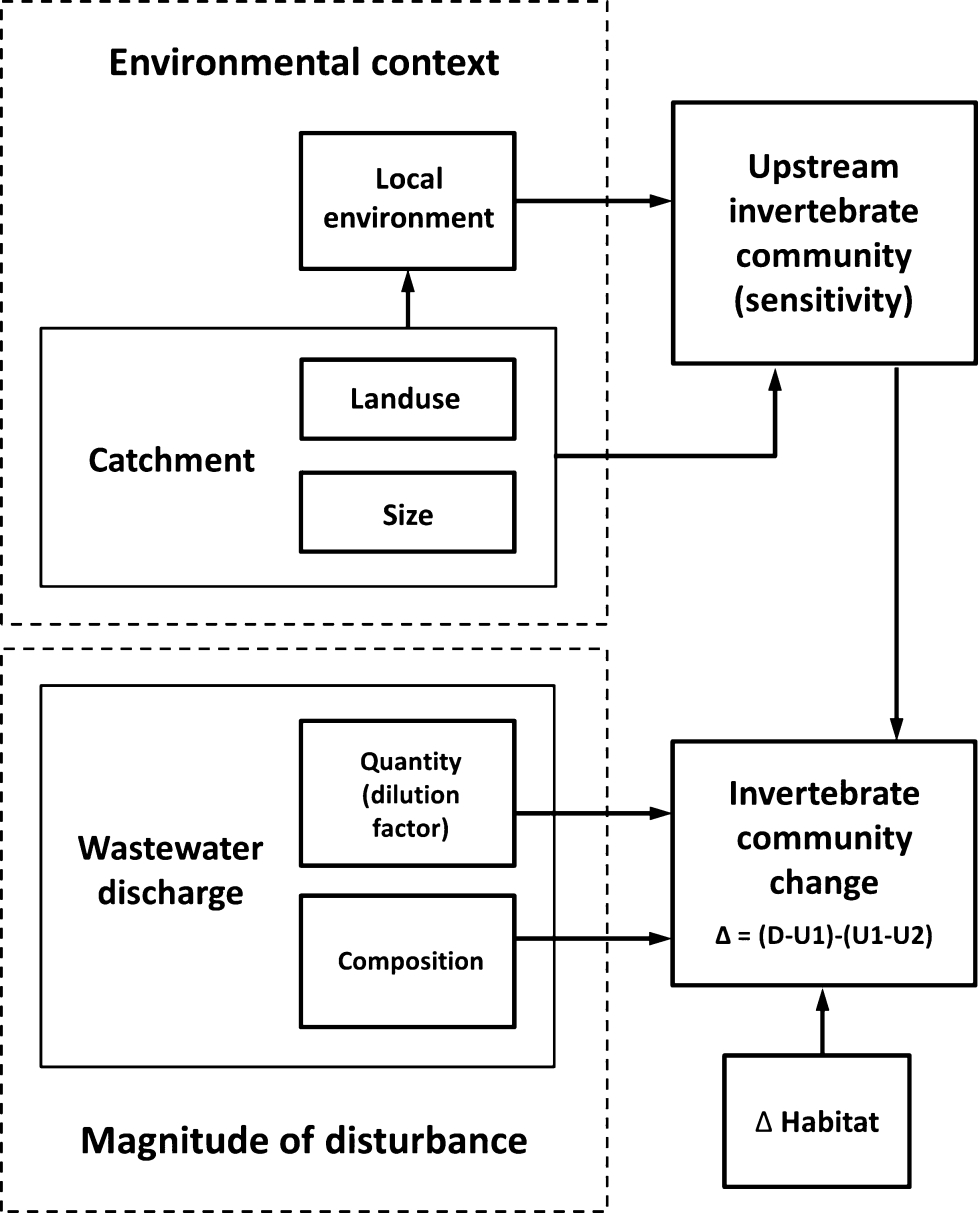
Conceptual diagram showing key variables influencing invertebrate community responses to wastewater inputs. This highlights the two hypotheses being tested: the importance of ‘environmental context’ (e.g., catchment land uses) versus the ‘magnitude of disturbance’ (i.e., wastewater inputs) on influencing invertebrate community change. Local environment refers to measurements taken from upstream reaches (e.g., water quality and habitat characteristics; see Table 1). Change (∆) was estimated from metrics at three sampling locations; two upstream (U1, U2) and the location (D) below the wastewater discharge (for further information see Methods).

We predicted that the environmental context (i.e., upstream anthropogenic pressures) would influence stream invertebrate community composition by filtering out pollution‐sensitive taxa (Heino 2013). These upstream influences (e.g., agricultural land uses) would mediate greater resistance to wastewater disturbances due to the presence of pollution‐tolerant communities (Allan 2004; Vinebrooke et al. 2004). We also predicted that the negative effects of wastewater on stream invertebrate communities should be greater with an increased magnitude of wastewater disturbance. Applying the concepts and terminology used in Lake (2000) and Poff et al. (1997), we defined wastewater inputs as a ‘press’ disturbance where the magnitude was greater in streams that receive more effluent relative to stream discharge. Thus, we hypothesized that:

1. the ‘magnitude of disturbance’ as a function of wastewater quantity (via dilution potential of the receiving habitat) and composition (chemical concentrations) would influence invertebrate community responses (Fig. 1). This hypothesis reflects Eq. (1) above, under the assumption that *∂Y*_*i*_ */∂X* is constant; and
2. the ‘environmental context’ would also strongly influence invertebrate community responses to wastewater disturbances (Fig. 1). Using Eq. (1), this hypothesis postulates that *∂Y*_*i*_ */∂X* varies as a function of upstream conditions *Y*_*i*_.

Our two main hypotheses are not mutually exclusive and our overall goal was to better understand the relative influence of ‘environmental context’ and the ‘magnitude of disturbance’ on community responses.

## Methods

### Site selection and study design

We selected 12 streams across Switzerland (Fig. A1, Appendix A) that are affected by inputs from wastewater treatment plants (WWTPs). To help avoid confounding influences, sites were selected so that no other WWTPs were located upstream, and had a minimum contribution of ~20% wastewater downstream of the WWTPs during low flow conditions (*Q*_347_, see below). The selected WWTPs varied in dilution potential and treatment technology, resulting in different levels of nutrient removal and subsequent pollution to receiving streams (Tables C1–2, Appendix C). The study streams were small to medium sized, with catchment areas ranging from 7.6 to 98.7 km^2^. Catchment areas were estimated from the dataset “GAB‐EZGG‐CH” (www.bafu.admin.ch/wasser/13462/13496/15009). Catchments were distributed across three Swiss biogeographical regions (Swiss Plateau, Jura, and Pre‐alps). The chosen catchments differed considerably in land‐use composition (ranges of areal extent for: forest, 9–46%; pasture, 5–60%; arable cropping, 0–52%; urban, 4–20%) and elevation (370–912 m a.s.l.). Land‐use data (“Arealstatistik 2009”) were obtained from Swiss land‐use statistics collected from 2004–2009 (www.landuse-stat.admin.ch). For further details regarding site characteristics, see Table A1, Appendix A.

At each study site, we chose one downstream sampling location (*D*) as an impacted site, and two upstream sampling locations (*U*1, *U*2) as reference sites (Fig. A2, Appendix A). Importantly, we used the two upstream reference sites to help quantify natural variation between sampling locations (i.e., the change between two sites without an additional pressure, akin to a ‘null’ model). This approach greatly strengthened the power of our inference when considering the downstream influence of wastewater (WW) at the impacted location (*D*). Sampling location *D* in each stream was positioned so that discharged WW was completely mixed across the wetted stream channel during low flow conditions. At each site, upstream and downstream reaches were as similar as possible with regard to stream morphology, riparian land use and vegetation. The range of distances between location *D* and WW inputs was 118–711 m. Distances between WW inputs and *U*1 and *U*2 locations ranged from 67 to 430 m and from 119 to 824 m, respectively. For further details regarding site selection and study design, see Appendix A.1.

### Physicochemical measurements

At each sampling location, we took monthly grab samples at baseflow conditions to measure 20 water quality parameters (Table 1). Sampling started in March 2013 for downstream (*D*) and upstream sites (*U*1), and in April 2013 for the most upstream (*U*2) locations, and continued until February 2014. Wastewater samples were collected on two sampling dates (June 2013 and February 2014). All water samples were collected in 1 L glass bottles, and transported on ice before storage at 4°C in the laboratory until analysis. Analyses of water chemistry were performed within 24 h. These analyses used standard methods described for the Swiss National River Monitoring and Survey Programme (NADUF; www.bafu.admin.ch/wasser/13462/14737/15108/15109).

**Table 1:** Summary of physicochemical variables used in analyses. See Methods for more details

Category	Group	Description
Water quality	Water	Conductivity (*μ*S/cm 20 °C), pH, alkalinity, hardness (mmol/L)
	Nutrients	${\text{NH}}_{4}^{+}$, ${\text{NO}}_{2}^{-}$, total and soluble reactive phosphorus (*μ*g/L), ${\text{NO}}_{3}^{-}$, total nitrogen, ${\text{SiO}}_{4}^{4-}$‐Si (mg/L)
	Major ions	Na^+^, K^+^, Ca^2+^, Mg^2+^, Cl^−^, ${\text{SO}}_{4}^{2-}$‐S (mg/L)
	Matter	Total and dissolved organic matter, total suspended solids (mg/L)
Habitat characteristics	Inorganic	Bedrock, boulder, cobble, gravel, sand, mud (%), Total and inorganic suspendible sediment (kg/m^2^)
	Organic	Coarse particulate organic matter, algae, bryophytes, submerged macrophytes, emergent macrophytes (%), organic suspendible sediment (kg/m^2^ and %)
	Hydromorphology	Flow (m/s), width, depth (m)

The ‘magnitude of disturbance’ consisted of two components: wastewater quantity and composition (Fig. 1). The wastewater quantity component was calculated as the dilution factor DF_*ww*_ (Eq. (2)), following Keller et al. (2014):

(2) $${\text{DF}}_{ww}=\frac{Q_{347}}{Q_{ww}}$$

where *Q*_347_ is the stream discharge that is reached or exceeded 347 days per year averaged over 10 years (equivalent to 95% of the time) and *Q*_*WW*_ is the annual mean discharge of wastewater. See Table A1 (Appendix A) for the actual dilution factors. The wastewater composition component was estimated based on Principal Components Analysis (PCA) of 20 water chemistry parameters (Table 1). These were summarized into one index (PC_*ww*_) using the PC1 scores, which accounted for 36% of the total variance (Table C3, Appendix C). DF_*ww*_ and PC_*ww*_ were not co‐linear (Pearson's product‐moment correlation, *r *= 0.27, *P *= 0.40).

At each sampling location, water depth and flow velocity (FlowTracker Handheld ADV, Sontek/YSI, Inc., San Diego, CA) was recorded at 10 equidistant points across three transects (also measuring wetted channel width) during low flow conditions in Autumn 2013. Benthic habitat composition was assessed visually at the time of invertebrate collections (see below) using standard Swiss protocols (Stucki 2010). Total benthic suspendible sediment (TSS; kg/m^2^) and derivations were recorded in Autumn 2013 (Clapcott et al. 2011). For more details on the methods used for TSS, see Appendix B.1. Change in habitat similarity below the wastewater discharge was estimated from habitat variables (Table 1) using PCA. The change in PCA site scores from three components was decomposed into one site score (PC_habitat_), explaining 45% of the variation in instream habitat (for more information, see Appendix B.3.3.2; Table C5, Appendix C).

### Benthic invertebrates

The invertebrate sampling methods followed standard protocols for benthic macroinvertebrate biomonitoring in Switzerland (Stucki 2010). At each sampling location (D, U1, U2), benthic invertebrates were sampled in Spring 2013 using a kick‐net (25 cm × 25 cm opening, 500‐*μ*m mesh size). Sites were sampled 13 March – 24 April 2013 with lower elevation sites (m a.s.l.) sampled first (Stucki 2010). Eight ‘kick‐net’ samples were collected to cover all major microhabitats found within each reach. All reaches had equal sampling effort (i.e., the number of samples collected and the duration of ‘kick‐net’ sampling), but to allow for the potentially greater number of microhabitats in larger streams, the area from which samples were selected was scaled proportional to the stream width (an area given by the mean wetted channel width^2^ × 10). The samples collected from each reach were pooled and stored in 80% ethanol prior to identification at a taxonomic level commonly used for biomonitoring in Switzerland (e.g., families) using predefined identification literature (Stucki 2010).

To describe invertebrate communities, we focused on four diversity (taxa richness, rarefied richness, Pielou's evenness, and Fisher's alpha) and two trait‐based indices (Saprobic Index and SPEAR Pesticides Index). We also used nonmetric multidimensional scaling (NMDS) scores (from two axes) to derive an index of total community change (see below). We recorded seven additional invertebrate community descriptors (total abundances, abundances minus oligochaetes, EPT family richness, relative abundances of EPT (%), %EPT minus oligochaetes, the Swiss IBCH biotic index, and the Berger‐Parker Index). These are closely related to the first six indices we focused on, meaning we did not consider them for our main analyses. Their results are shown in Table C6, Appendix C.

The Saprobic Index (SI) ranges between 1 and 4, and increases with greater amounts of easily degradable organic material, indicating shifts in the invertebrate community toward taxa that are more tolerant of low oxygen conditions (Bunzel et al. 2013). The SPEAR Pesticide Index (SPEAR Index hereafter) describes the proportion of taxa (%) susceptible to pesticides. Lower relative abundances of SPEAR taxa indicate pesticide stress and it is used extensively as an index of stream health in Europe (Liess and Ohe 2005; Beketov et al. 2009). For more information on these indices and their calculation, see Appendix B.2.

### Data analysis

The data analysis section below first focuses on the ordination methods used to describe invertebrate community composition above and below the wastewater discharge. The purpose of these analyses was twofold. Firstly, we wanted to better understand how stressors associated with land use and wastewater influence stream communities. Secondly, we wanted to use these analyses to derive indices of community composition and change. Specifically, we wanted to derive an index of total community change (see NMDS below) in response to wastewater. This index provided an additional measurement of community change to the taxonomy and trait‐based descriptors described above. We also wanted to map upstream community composition with catchment land‐uses to provide independent predictor variables (see pRDA) to test our ‘environmental context’ hypothesis.

### Ordinations describing differences in invertebrate community composition

To contrast macroinvertebrate community composition above and below the wastewater discharge, we used unconstrained and constrained ordinations. We performed nonmetric multidimensional scaling (NMDS) analysis on Hellinger‐transformed relative abundance data using the Euclidean‐distance metric (Legendre and Gallagher 2001). This unconstrained analysis provided the basis for the index of total community change (see below). Permutational multivariate analysis of variance (PERMANOVA) was used on the same data to test for statistical differences in community composition between sampling locations. One study site (Kernenried) was omitted from the PERMANOVA analyses due to exceptionally high proportions of gammarid amphipods at all three sampling locations (Fig. C2, Appendix C). This did not materially affect the results of the tests, but did improve their explanatory power.

We used four partial redundancy analysis (pRDA) models to test the association of physicochemical parameters (land use, water quality and instream habitat) with invertebrate community composition after ‘partialing’ out the effects of spatial location (i.e., spatial structuring leading to autocorrelation of communities). The first pRDA model tested environmental determinants and spatial influences on upstream communities only (*U*1, *U*2). This analysis was also used to provide independent predictor variables to test our ‘environmental context’ hypothesis. The second pRDA model tested environmental and spatial influences on communities from all three sampling locations (*D*,* U*1, *U*2), and was used to describe the potential stressors in wastewater influencing downstream communities. The other two pRDA models complemented these main analyses (for further information, see Appendix B.3.2.2).

In all pRDA models, we used Hellinger‐transformed relative abundance data to create the biological matrices (Legendre and Gallagher 2001). Similarly, in all models spatial structuring of communities using Cartesian coordinates was assessed using Principal Coordinates of Neighbours Matrices (PCNM) analysis (Borcard and Legendre 2002). To avoid over‐parameterizing the models, we first removed spatial and environmental variables that were co‐linear with other variables (*r* < 0.6) or unimportant using a forward selection procedure where the significance of each independent variation component was permutation tested using 1000 randomizations (Peres‐Neto et al. 2003).

In the upstream pRDA model, we tested the proportion of variation in community structure at upstream sites explained by catchment land‐uses (% area in upstream catchment two agricultural land‐use classes, ‘arable cropping’ and ‘pasture’), spatial location (five PCNM axes), and ‘reach‐scale’ environmental predictors (concentrations of ammonia, soluble reactive phosphorus, nitrate, specific conductivity, dissolved organic carbon, suspended sediment, and average water velocities). The site scores from the first three axes of this upstream pRDA model (where spatial location was “partialed” out) were used as independent predictor variables in multiple‐regression models testing our main hypotheses (see below).

To account for the influence of wastewater effects at downstream sites, we used a second pRDA model for all sampling locations (*D*,* U*1, *U*2). We partitioned the variation in community structure explained by the stream (i.e., ‘catchment’), spatial location (one PCNM axis), and the ‘reach‐scale’ environmental predictors (concentrations of ammonia, soluble reactive phosphorus, sodium, nitrate, and dissolved organic carbon; % cover of submerged macrophytes; % organic benthic suspendible sediment; and specific conductivities) which were attributed to the influences of wastewater. We have presented the results of this model with stream and spatial location “partialed” out (Fig. 3B).

For these analyses, we used the R package ‘vegan’ (Oksanen et al. 2010) with functions ‘metaMDS’ for NMDS, ‘adonis’ for PERMANOVA, and ‘rda’ for partial redundancy analyses with ‘varpart’ for variation partitioning.

### Testing our main hypotheses: environmental context and magnitude of disturbance

To test the ‘environmental context’ and ‘magnitude of disturbance’ hypotheses, we used a total of seven invertebrate community descriptors as response variables (taxa richness and its rarefied equivalent, evenness, Fisher's alpha, Total Community Change, and the Saprobic and SPEAR indices; Table 2).

**Table 2:** Summary of response and predictor variables used in multiple‐regression models testing our two hypotheses ‘environmental context’ and ‘magnitude of disturbance’. Response variables were derived from stream invertebrate community data. Changes refer to differences between upstream and downstream (wastewater‐impacted) sampling locations. NMDS, Non‐metric multidimensional scaling; pRDA, Partial redundancy analysis; PCA, Principal components analysis; US, Upstream. See Methods for more details

Type	Group	Variable	Description
Response	Invertebrates	∆ Taxa	Change in taxa richness
		∆ Rarefied	Change in rarefied taxa richness
		∆ Evenness	Change in taxa evenness (Pielou's J)
		∆ Fisher's	Change in rare taxa (Fisher's *α*)
		∆ Community	Change in community similarity (NMDS)^a^^,^ b
		∆ SPEAR	Change in SPEAR Index
		∆ Saprobic	Change in Saprobic Index
Predictor	‘Context’	RD1_invert_	US invertebrates (pRDA Axis 1)^a^
		RD2_invert_	US invertebrates (pRDA Axis 2)^a^
		RD3_invert_	US invertebrates (pRDA Axis 3)^a^
	‘Disturbance’	DF_*ww*_	Wastewater quantity (Dilution factor)
		PC1_*ww*_	Wastewater composition (PCA Axis 1)
	Habitat	PC_habitat_	Change in habitat similarity (PCA)

a Community composition using relative abundance data.

b Total Community Change.

For the six diversity and trait‐based response variables (taxa richness and its rarefied equivalent, evenness, Fisher's alpha, and the Saprobic and SPEAR indices), we compared the (directional) differences between the *D* and *U*1 sampling locations to the differences between the two upstream locations (Eq.(3)) as:

(3) $$\text{Community descriptor}\;{\text{change}}_{i}=\left(D_{i}-U1_{i}\right)-\left(U1_{i}-U2_{i}\right)$$

where *i* is the study stream, *U*1 and *U*2 are the upstream location, and *D* is the downstream location.

To calculate the total community change index, we used the NMDS scores from two axes. The difference in NMDS scores between downstream site (*D*) and upstream site (*U*1; Change, Eq.(4)) was corrected using the difference between the two upstream sites (i.e., community change between two unaffected sites; Null Change, Eq.(5)) to provide Total Community Change (Eq.(6)):

(4) $${\text{Change}}_{i}=\sqrt{{{\left(\text{NMDS}1_{D}-\text{NMDS}1_{U1}\right)}_{i}}^{2}+{{\left(\text{NMDS}2_{D}-\text{NMDS}2_{U1}\right)}_{i}}^{2}}$$

(5) $$\text{Null}\;{\text{change}}_{i}=\sqrt{{{\left(\text{NMDS}1_{U1}-\text{NMDS}1_{U2}\right)}_{i}}^{2}+{{\left(\text{NMDS}2_{U1}-\text{NMDS}2_{U2}\right)}_{i}}^{2}}$$

(6) $$\text{Total Community}\;{\text{Change}}_{i}={\text{Change}}_{i}-\text{Null}\;{\text{change}}_{i}$$

where *i* is the study stream, *U*1 and *U*2 are the upstream locations, and *D* is the downstream location.

### Predicting changes in invertebrate community descriptors

For each of the seven response variables described above, we used six predictor variables in the multiple‐regression models (Table 2):

1‐3 upstream community composition (reflecting the ‘environmental context’ hypothesis). Using the *U*1 site scores from the upstream pRDA model where spatial influences were “partialed” out, the first three axes (RD1_invert_, RD2_invert_, and RD3_invert_) represented different dimensions of upstream community composition (Fig. 1);
4 the wastewater dilution factor DF_*ww*_ (Eq. (2)), describing the wastewater quantity component of the ‘magnitude of disturbance’ hypothesis (Fig. 1);
5 wastewater composition using the PC1 scores (PC_*ww*_) as another component of the ‘magnitude of disturbance’ hypothesis (Fig. 1); and
6 the change in physical habitat between upstream and downstream sites (PC_habitat_). This was likened to an ‘error term’, accounting for any instream habitat variation between sampling locations (Fig. 1).

We used hierarchical partitioning in the multiple‐regression models. This approach is frequently used to evaluate the independent effects of predictor variables on their correlation with responses (Burdon and Harding 2008). This approach used *R* ^2^ values to determine the proportion of variance explained independently and jointly by variables, where all possible models in a multiple‐regression setting are considered. A total of 1000 randomizations of the data matrix allowed the quantification of relative ‘effect sizes’ associated with the partitioning by estimating the *Z*‐score for each predictor variable (Mac Nally 2000). Statistical significance was based on the upper 95% confidence limit (*Z*‐score > 1.65); analyses were conducted using the ‘hier.part’ package in R (Walsh and Mac Nally 2007). Additionally, we calculated univariate regressions and partial correlations (using the ‘ppcor’ package in R) where appropriate to show the response nature. For more details on these data analyses, see Appendix B.3.

### Mixed‐models describing differences in invertebrate community descriptors

We used analysis of variance (ANOVA) with linear mixed‐effects (LME) models to test for the general response of stream invertebrate communities to wastewater using the same community descriptors described above. LME models accounted for the random effect of stream while testing for the fixed effect of sampling location (*D*,* U*1, *U*2). LME models were generated with the R package ‘lmer4’. We identified post‐hoc differences using a least‐squares means approach with multiplicity adjustments (Tukey's HSD) obtained from the R package ‘lsmeans’. The variance explained by the random effect (streams) was calculated using the R code provided in Nakagawa and Schielzeth (2013). Mean standardized effect sizes (Cohen 1992) were calculated between *D* and upstream controls (*U*1, *U*2) to show the relative magnitude of differences. For further details on effect sizes, see Appendix B.3.1. All analyses were conducted in R (R Development Core Team (2013).

## Results

### Variation in upstream environmental context across streams

The water quality data from upstream locations showed clear influences of catchment land use. Mean dissolved phosphorus concentrations ranged from 5.0 to 51.6 *μ*g/L and mean nitrate from 0.8 to 6.4 mg/L among streams at upstream sampling locations. Therefore, based on Swiss assessment protocols (Liechti 2010), almost 50% of the streams at the upstream locations could be classified as ‘moderate’ to ‘very bad’ based on phosphorus, but all except one stream represented ‘good’ chemical status based on nitrogen. Water quality was probably reduced by agricultural activities; dissolved phosphorus was positively correlated with increasing catchment land cover of arable cropping and pasture (*F*_1,10_ **= 5.98, *R* ^2^ **= 0.37, *P* < 0.05), and nitrate levels with arable cropping land cover alone (*F*_1,10_ **= 17.3, *R* ^2^ **= 0.63, *P* < 0.01). For more information on upstream water quality, see Appendix C.1, Table C1.

Invertebrate communities at upstream sampling locations were generally dominated by chironomid dipterans, baetid mayflies, and gammarid amphipods (Table C7, Appendix C). Applying Swiss assessment protocols to the upstream locations indicated that on average, nine of the streams achieved ‘good’ status, with three being ‘moderate’ (Stucki 2010). For a full description of common and abundant taxa found at study sites, see Appendix C.4.1.

Invertebrate community data from upstream locations showed the influence of catchment land uses and water quality. Independently, environmental variables including eight water chemistry parameters (concentrations of nitrate, ammonia, phosphorous, dissolved organic carbon, suspended sediment, and specific conductivity) and mean flow velocity explained 43% (Variation partitioning, ‘varpart’, *F*_7,13_ **= 5.39, *P* < 0.01) of the variation in upstream invertebrate community composition. Spatial separation of sites alone accounted for 16% of variation (‘varpart’, *F*_1,13_ **= 5.10, *P* < 0.01) while catchment land uses (% arable cropping and pasture) explained 6% (‘varpart’, *F*_2,13_ **= 2.19, *P* < 0.05). The joint variation of catchment and local environment explained another 3%.

**Figure 2:**
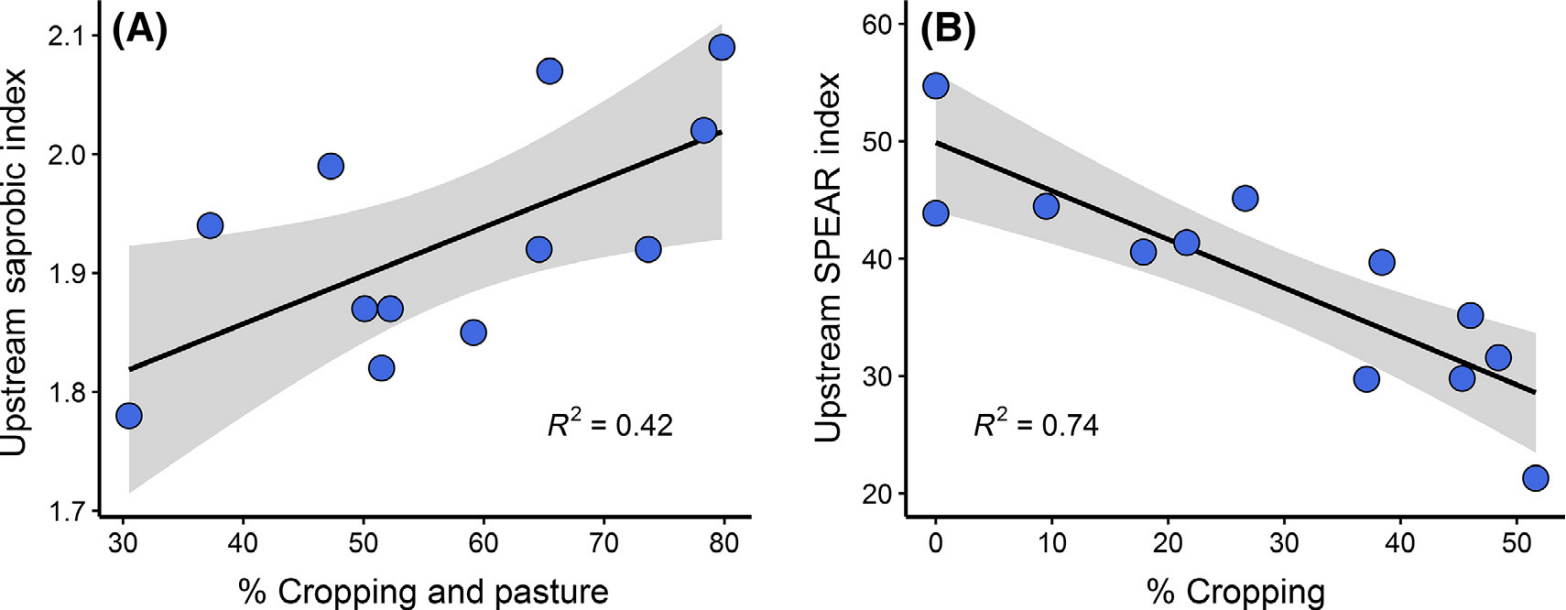
Linear regressions of upstream invertebrate community descriptors and catchment agriculture: A) mean upstream Saprobic Index scores and agriculture (% catchment area used for arable cropping and pasture), and B) mean upstream SPEAR Index scores and arable cropping (% catchment area). Shaded areas indicate 95% confidence intervals.

None of the diversity indices were significantly associated with human land use or catchment size. At upstream sites, the Saprobic Index increased with agricultural land cover (arable cropping and pasture; *F*_1,10_ **= 7.19, *R* ^2^ **= 0.42, *P* < 0.05, Fig. 2A) and the SPEAR Index decreased with cropping land cover (*F*_1,10_ **= 27.8, *R* ^2^ **= 0.74, *P* < 0.001, Fig. 2B). These results indicate that an increased extent of agricultural land uses in the catchment results in increased abundances of saprobic‐tolerant taxa and reductions in taxa sensitive to pesticide pollution.

RD3_invert_ from the redundancy analysis of upstream community composition correlated positively with upstream saprobic condition (*F*_1,10_ **= 15.5, *R* ^2^ **= 0.61, *P* < 0.05, Fig. 4A), indicating that this descriptor of upstream community composition was associated with abundances of saprobic‐tolerant taxa (e.g., oligochaete worms). In contrast, the SPEAR Index was best described by scores from the first three axes of the upstream redundancy analysis of community composition (RD1_invert_, RD2_invert_, RD3_invert_), meaning it was not possible to distinguish a pRDA‐derived index for the SPEAR taxa. For more results on upstream invertebrate community composition, see Figure C1, Appendix C; for further descriptions of environmental context, see Appendix C.2.

### Characteristics of wastewater disturbance across streams

Wastewater inputs further impaired streams by contributing nutrients, major ions, and organic matter (Table C2, Appendix C). The fraction of wastewater downstream of WWTPs ranged from 23–133% of upstream discharge (Q_347_) among sites (Table A1, Appendix A). Among sites, we observed ranges of 7.5–30.2 mg/L for mean nitrate and 24–2786 *μ*g/L for mean dissolved phosphorus (see Table C2, Appendix C for more information). These differences in WW amount and composition may reflect different WW treatment technologies. The coefficient of variation for the magnitude of WW disturbance and upstream environmental context (i.e., agricultural land cover) were similar, suggesting that the gradient lengths of these predictors were comparable in scope. For more information, see Appendix C.2.

### Community responses to wastewater disturbances: diversity and composition

Invertebrate community diversity declined downstream of wastewater inputs (Table 3). Although taxa richness did not change significantly, both its rarefied equivalent and community evenness decreased downstream. Fisher's alpha was significantly lower downstream, indicating a loss of rare taxa to wastewater disturbances (Table 3). Results from the seven additional descriptors we calculated can be viewed in Table C6, Appendix C. Briefly, there were no significant differences in the Swiss IBCH biotic index, suggesting that this descriptor is insensitive to wastewater impacts. Similarly, there was no difference in EPT taxa richness (family level) which may reflect the taxonomic resolution used. EPT relative abundances were significantly lower downstream, but this was not significant when the oligochaete worms were excluded. Raw abundances of all invertebrates increased downstream due to the positive response of oligochaetes to wastewater, which also contributed to greater dominance (Berger‐Parker Index).

**Table 3:** Taxonomic and trait‐based indicators of stream condition at sampling locations downstream (*D*) and upstream (*U*1, *U*2) of wastewater inputs using macroinvertebrate community data collected from 12 Swiss streams sampled during Spring 2013. Mean values are presented ±1 standard deviation. Cohen's *d* quantifies differences in invertebrate community metrics downstream (*D*) compared to upstream (*U*1, *U*2). F‐statistics, degrees of freedom, *P*‐values, and the proportion of variance explained by the random factor (Stream) are presented from mixed‐model ANOVAs where the sampling location (*U*1, *U*2, *D*) was the fixed factor

Community approach used	Indicator	Sampling location			Cohen's *d*	*F‐*stat	df	Significance	Stream % var.
		*U*2	*U*1	*D*					
Taxonomic	Taxa richness	25.1 ± 5.9	24.8 ± 5.4	24.3 ± 6.0	−0.12	1.37	2, 24	ns	95
	Rarefied richness	21.0 ± 4.0	20.9 ± 4.6	17.8 ± 4.6	−0.68	19.9	2, 22	*P* < 0.001	80
	Evenness	0.57 ± 0.08	0.57 ± 0.09	0.45 ± 0.11	−1.07	23.2	2, 22	*P* < 0.001	51
	Fisher's alpha	4.07 ± 1.03	4.03 ± 1.12	3.59 ± 0.99	−0.44	5.95	2, 22	*P* < 0.01	84
Trait	Saprobic Index	1.91 ± 0.09	1.95 ± 0.13	2.39 ± 0.21	1.60	37.4	2, 33	*P* < 0.001	0^a^
	SPEAR Index	38.1 ± 9.0	38.1 ± 9.1	34.2 ± 8.9	−0.44	13.9	2, 22	*P* < 0.001	91

a Although the mixed‐model indicated that streams explained 0% variance of the Saprobic Index, a linear‐model suggested 4.5%.

The magnitude of diversity changes between upstream and downstream sites did not show consistent patterns with environmental context or disturbance (Table C9, Appendix C). However, changes in taxa richness may have decreased with the amount of habitat change (PC1_habitat_), indicating that the creation of new niches by differences in habitat may confound diversity responses to wastewater (Partial correlation, ‘ppcor’, *r *= −0.42; Hierarchical partitioning, ‘hier.part’, *Z *= 1.75, *P* < 0.05; Table C9, Appendix C).

**Figure 3:**
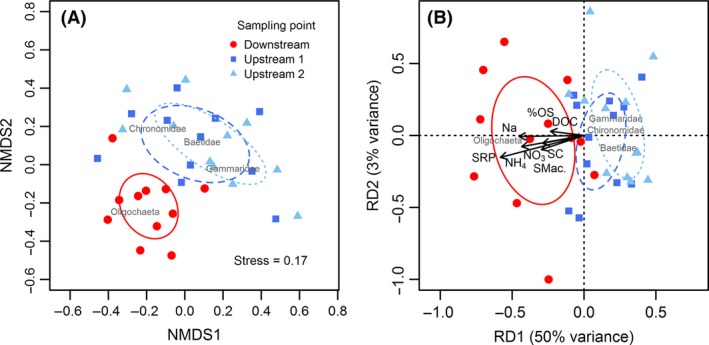
Invertebrate community relative abundance data analysed using A) nonmetric dimensional scaling (NMDS); B) partial redundancy analysis (pRDA). Standard dispersion ellipses represent 95% confidence intervals. Common invertebrate taxa shown. NH_4_, ammonia (*μ*g/L); SRP, soluble reactive phosphorus (*μ*g/L); Na, sodium (mg/L); %OS, organic suspendible sediment (% total); NO_3_, nitrate (mg/L); SC, specific conductivity (*μ*S/cm 20° C), DOC, dissolved organic carbon (mg/L); SMac., submerged macrophytes (% cover).

**Figure 4:**
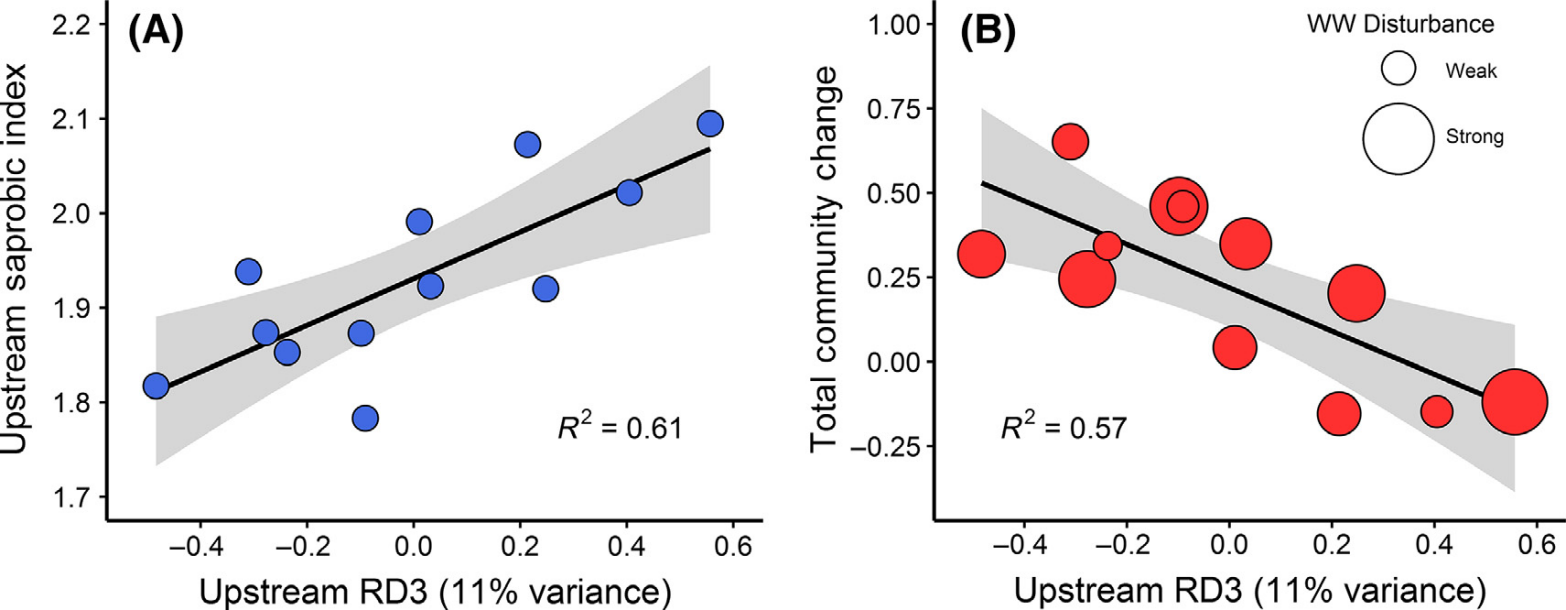
Linear regressions of upstream community composition (Axis 3 scores from the upstream pRDA model) and A) mean upstream Saprobic Index and B) Total Community Change (NMDS‐derived) at downstream impacted sites. Values at sites D (in red) are scaled according to the magnitude of wastewater disturbance. Shaded areas indicate 95% confidence intervals.

**Figure 5:**
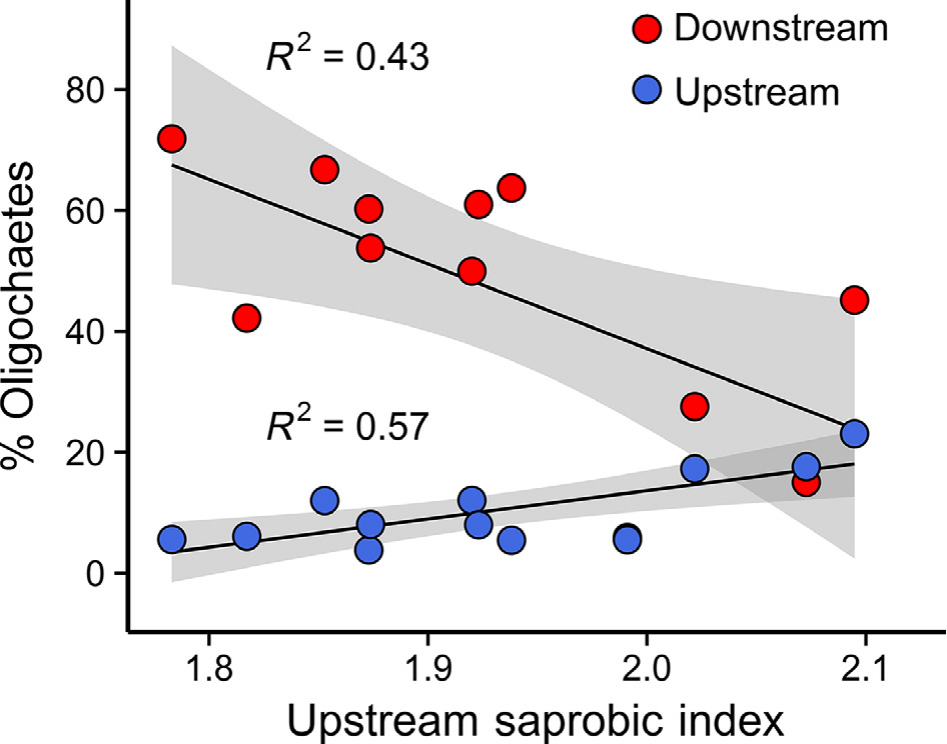
Linear regressions of mean upstream Saprobic Index scores and relative abundances of oligochaete worms at sampling locations upstream (U1, U2) and downstream (D) of a wastewater discharge. Mean abundances of oligochaetes are shown for upstream sites U1 and U2. Shaded areas indicate 95% confidence intervals.

Invertebrate community composition changed downstream (NMDS, Fig. 3A; ‘adonis’, *F*_2,32_ **= 5.49, *R* ^2^ **= 0.26, *P* > 0.001). Total Community Change was strongly associated with upstream community composition (RD3_invert_, Fig. 4B; *F*_1,10_ **= 15.3, *R* ^2^ **= 0.61, *P* < 0.01; ‘ppcor’, *r *= −0.85; ‘hier.part’, *Z *= 4.39, *P* < 0.05; Table C9, Appendix C). This partly reflected the association of RD3_invert_ with saprobic‐tolerant taxa such as the oligochaete worms, which in turn strongly influenced downstream community composition. Increased relative abundances of oligochaetes upstream meant less community change downstream in response to wastewater (see also Fig. 5). Chironomid dipterans, gammarid amphipods, and baetid mayflies were relatively more abundant upstream, and abundances of oligochaete worms strongly increased downstream (Fig 3A; Table C7, Appendix C). When oligochaetes were excluded from the NMDS analyses, the differences between upstream and downstream communities were not statistically significant (Fig. C3A, Table C8, Appendix C.4.2).

Several water quality parameters (i.e., phosphorous, nitrate, ammonia, sodium, dissolved organic carbon, and specific conductivity) and two indicators of instream habitat (i.e. organic % of benthic fine sediments and reduced macrophyte cover) increased with wastewater discharge and appeared to influence downstream community structure (Fig. 3B). These environmental factors independently explained 17% of the variation in community composition (‘varpart’, *F*_8,15_ **= 3.04, *P* < 0.01) after ‘partialing’ out stream and spatial location (i.e., one PCNM axis).

### Community responses to wastewater disturbances: trait‐based indices

**Figure 6:**
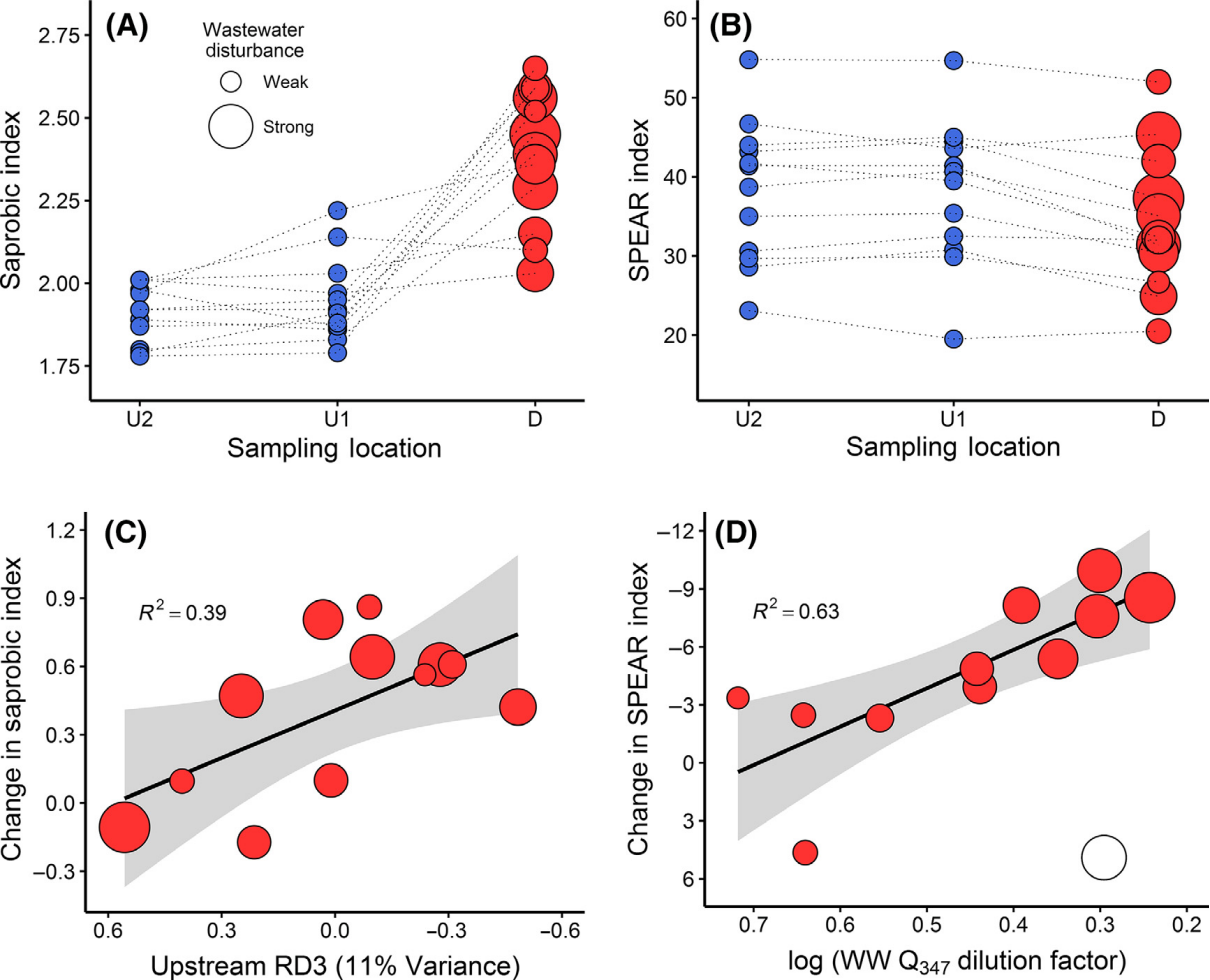
Changes in two trait‐based invertebrate community descriptors: A) The Saprobic Index scores increased at sites D below the wastewater discharge relative to upstream controls (U1, U2), whereas B) the SPEAR Index decreased at sites D. C) Community change (sensitivity) using the Saprobic Index scores was correlated with upstream community composition (RDA Axis 3 scores). In contrast, D) the change in the SPEAR Index scores was correlated with the magnitude of wastewater disturbance (Q_347_ dilution factors). Values at sites D (in red) are scaled according to the magnitude of wastewater disturbance and axes are reversed for consistency. Shaded areas indicate 95% confidence intervals. The black circle in Fig. 2D is an outlier; for more information, see Appendix C.4.2.6.

Both the Saprobic and SPEAR Indices changed downstream (Table 3), but effect sizes suggested that wastewater inputs had greater effects on organic enrichment (Saprobic Index) than on pesticide toxicity (SPEAR Index). The negative change in the SPEAR Index (i.e., reductions in relative abundance of SPEAR taxa) was significantly associated with the wastewater dilution factor (DF_*WW*_; Fig 6B and D; *F*_1,9_ **= 12.9, *R* ^2^ **= 0.63, *P* < 0.01; ‘ppcor’, *r *= −0.92; ‘hier.part’, *Z *= 4.54, *P* < 0.05; Table 4). This result suggests that larger fractions of wastewater resulted in greater reductions of sensitive taxa. This was despite the wide range of SPEAR values at the upstream locations (e.g., 21–55%), thus implying that community sensitivity to pesticides and pesticide‐like compounds was not context dependent.

**Table 4:** Results from multiple regression models of invertebrate trait‐based descriptor change responses using hierarchical partitioning. Shown are the independent (*I*), joint (*J*) and total effects of predictors on the response variables. I% represents the contribution of the *I*‐values to the total explained variance in the response variables. The partial correlation coefficient (pcor) indicates the nature of the relationship between significant predictor variables (from hierarchical partitioning) and the response variable, after ‘partialing’ out other predictors. For a full description of response and predictor variables, see Table 2

Response	Predictor	*I*	*J*	Total	%*I*	Obs	*Z*‐score	pcor
∆ Saprobic	RD3_invert_	0.450	−0.038	0.412	72.0	0.45	3.13^a^	−0.73^b^
	RD1_invert_	0.064	0.036	0.101	10.3	0.06	−0.33	
	PC_habitat_	0.044	−0.042	0.002	7.1	0.04	−0.57	
	RD2_invert_	0.035	−0.022	0.013	5.6	0.04	−0.62	
	PC1_*ww*_	0.016	0.012	0.029	2.6	0.02	−0.81	
	DF_*ww*_	0.015	−0.015	0.000	2.4	0.01	−0.82	
∆ SPEAR	DF_*ww*_	0.534	0.094	0.628	58.4	0.53	4.54^a^	−0.92^b^
	RD1_invert_	0.186	0.134	0.320	20.4	0.19	0.76	
	PC_habitat_	0.142	0.137	0.279	15.5	0.14	0.43	
	RD2_invert_	0.021	0.004	0.025	2.3	0.02	−0.80	
	RD3_invert_	0.018	−0.003	0.015	1.9	0.02	−0.82	
	PC1_*ww*_	0.013	0.004	0.017	1.4	0.01	−0.87	

a Based on the upper 95% confidence limit (*Z* > 1.65).

b *P < *0.05.

Although the Saprobic Index (SI) change from upstream to downstream was not related to DF_*ww*_, the presence of a wastewater input seemed to ‘trigger’ an inverse response to upstream organic enrichment (Fig. 5). The relative abundance of oligochaete worms at upstream (*F*_1,10_ **= 13.3, *R* ^2^ **= 0.57, *P* < 0.01) and downstream (*F*_1,10_ **= 7.62, *R* ^2^ **= 0.43, *P* < 0.05) locations was significantly correlated with upstream saprobity (Fig. 5). Interestingly, the slopes differed (Fig. 5), with downstream oligochaetes (*b*_*D*_ **= −140.0) showing a contrasting pattern to upstream oligochaetes (*b*_*U*_ **= 46.8), indicating that the extent of change between upstream and downstream was larger when conditions were less polluted upstream (i.e., lower SI).

Similarly, the positive downstream change in the SI was correlated with upstream community composition based on RD3_invert_ (Fig 6A and C; *F*_1,10_ **= 6.3, *R* ^2^ **= 0.39, *P* < 0.05; ‘ppcor’, *r *= −0.73; ‘hier.part’, *Z *= 3.13, *P* < 0.05; Table 4). This indicates that less eutrophic streams (i.e., sites with low SI scores) were more sensitive to wastewater inputs than more degraded sites (i.e., streams with higher SI scores).

## Discussion

One challenge for community ecology is making general statements about ecological patterns, which are often contingent on the organisms involved and their environmental context (Lawton 1999). We predicted that upstream communities already influenced by land‐use pressures would not suffer further impairment by wastewater because of resistance to change conferred by environmental filtering (e.g., communities dominated by pollution‐tolerant taxa). Supporting the ‘environmental context’ hypothesis, we found that Saprobic Index and Total Community Change (NMDS‐derived) changes in response to wastewater inputs were correlated with upstream community composition, which in turn, was influenced by catchment land uses. We also predicted that the amount and composition of wastewater entering the receiving stream would determine the extent of change. Supporting the ‘magnitude of disturbance’ hypothesis, we found that the change in the SPEAR Index was correlated with the relative amount of wastewater discharged to the stream. Taken together, these results indicate that agricultural land uses can ‘mask’ the detection of wastewater impacts with regards to eutrophication (e.g., saprobity), but the widespread presence of pesticides (and pesticide‐like compounds) in treated effluent drives the correlation between ecological impacts (i.e., chemical toxicity) and wastewater concentrations in receiving environments.

### Context‐dependent responses to wastewater

Environmental context can reflect different ecosystem properties such as disturbance regime and habitat heterogeneity, and may be an important determinant of the relationship between community structure and function (Cardinale et al. 2000). Responses to disturbance (e.g., wastewater pollution) may be similarly contingent on the surrounding environment and resulting communities. For instance, Gücker et al. (2006) suggested that upstream habitat degradation and eutrophication leading to impoverished invertebrate faunas meant the adverse impacts of WWTPs on their streams were small when compared to other studies. These anthropogenic effects may have led to environmental filtering (akin to ‘species sorting’ in metacommunity ecology; Heino 2013), where only resistant taxa were left after prior exposure to pollution upstream. In our study, we found that Fisher's alpha was reduced at the downstream locations, indicating that rare taxa were lost from communities impacted by wastewater. However, wastewater‐induced changes in rare and SPEAR taxa were not influenced by upstream agricultural land uses. Although Total Community (NMDS‐derived) and Saprobic Index changes were environmentally contingent, it seems probable that the response of one pollution‐tolerant taxon (i.e., oligochaete worms) to wastewater strongly contributed to these correlations.

The increased abundances of oligochaetes (e.g., *Tubifex tubifex* Müller 1774) at downstream sites is an archetypal response to wastewater inputs in freshwaters (Hynes 1960). This reflects their tolerance of low levels of dissolved oxygen caused by organic enrichment (Aston 1973), but potentially also increased resource availability and a relative decrease in competition and predation (Martin et al. 2008). Interestingly, the increase in the relative abundance of oligochaetes at downstream locations was weaker when upstream conditions were more degraded (Fig. 5). This suggests that the direct effects of additional pollution may have reduced the increases in abundances of oligochaetes responding to wastewater inputs. Potentially, the additional toxicity stress dampened the positive influences of resource subsidies in the treated effluent (Singer and Battin 2007), where smaller than expected cumulative impacts of stressors can indicate antagonistic interactions (Folt et al. 1999). Alternatively, the indirect effects of pollution may have dampened oligochaete responses, where more pollution‐tolerant communities induced greater competition and predation, thereby masking the effects of wastewater inputs on community composition. Stress‐induced community tolerance can reduce the impacts of additional stressors through prior exposure and positive co‐tolerances (Vinebrooke et al. 2004).

The context dependent effects we observed on Total Community Change may reflect the taxonomy‐based approach used. Downstream change in communities using the NMDS analysis (Fig. 3A) derives from both positive and negative responses of taxa to wastewater inputs. Thus, in addition to the putative negative influences on more sensitive taxa, our Total Community Change index includes the increased abundances of tolerant taxa (e.g., oligochaete worms). This may reflect compensatory dynamics of communities that can obscure changes in population‐level responses to disturbance and environmental stress (Supp and Ernest 2014). Taxa richness is similarly affected by compensatory dynamics (Magurran 2016), but we did not find any evidence for either mechanism (loss and replacement) affecting beta diversity (Fig. C4, Appendix C). Instead, stochastic influences of dispersal such as mass effects (Heino 2013) may have affected the presence of taxa at downstream locations due to drift from upstream sites.

Importantly, we used a relatively coarse taxonomic resolution in our study (e.g., family level), which may have obscured changes apparent at higher levels of taxonomic resolution. Species‐level data can be useful because species may have different niche requirements (Cranston 1990). However, environmental assessments based on higher‐level taxonomy are prone to errors due to misidentifications (Schmidt‐Kloiber and Nijboer 2004). In contrast, family‐level abundance data are recommended as the best resolution for resolving patterns in macroinvertebrate assemblages and ranking sites according to their conservation value (Marshall et al. 2006; Heino and Soininen 2007). In an area similar to our study region, Beketov et al. (2009) found that the explanatory power of the family‐level SPEAR index was not significantly lower than the species‐level equivalent.

Another possible explanation for why we saw less community change in more degraded habitats is that the tolerance of exposed populations may have increased due to physiological adaptation or through adaptive genetic responses to natural selection (Reznick and Ghalambor 2001). However, local adaptation to pollution may be weak if chronic stress levels lead to small populations and/or there is high gene flow between different sites (Rolshausen et al. 2015), which may be the case in the stream ecosystems we studied. Although it is highly likely that both ecological and evolutionary processes determine community structure, we cannot disentangle these factors as we only assessed structural differences in communities, not phenotypic or genetic changes in responding taxa.

### Response to the magnitude of wastewater disturbance

In contrast with the Saprobic Index, the response of the SPEAR Index to wastewater was not environmentally contingent, but instead associated with a component of the ‘magnitude of disturbance’ hypothesis. Wastewater dilution factors appeared to influence the change in the SPEAR Index, suggesting the widespread presence of pesticides or pesticide‐like compounds in effluents. This may have explained why we were unable to detect an influence of wastewater composition, because we did not use measured micropollutant data in our analyses. Pesticides can be significant constituents of wastewater effluent and may have spatial ‘legacy effects’ that extend below the discharge (Wittmer et al. 2010; Bunzel et al. 2013). We used streams in catchments where agriculture was the dominant land use, and it is plausible that the presence of arable land with associated uses of pesticides leads to greater concentrations of these contaminants entering untreated wastewater effluents.

We defined the discharge of wastewater at each site as a single pressure. However, wastewater effluent is a complex mixture of multiple stressors that includes nutrients, organic matter, toxicants, and pathogens (Appendix C, Table C2; Wittmer et al. 2010; Eggen et al. 2014). Increased organic enrichment and decreased dissolved oxygen availability (i.e., saprobity) has long been known as a major factor influencing stream ecosystems (Hynes 1960). More recently, heavy metals (Stalter et al. 2013) and organic toxicants, such as pesticides (Bunzel et al. 2013), have been identified as potential drivers of ecological change due to inputs of wastewater. The mixture of toxicants, together with other physicochemical changes exerted by treated effluent on receiving habitats (e.g., lower dissolved oxygen, altered thermal regimes) makes attributing causality to any one stressor difficult. The reduction of micropollutant loads in discharged wastewater through treatment upgrades provides one avenue to better understand causal pathways (Eggen et al. 2014). However, there is a need for experimental manipulations that disentangle different stressors present in treated effluent to better understand their relative importance and possible interactive effects.

### Multiple pressures and nonlinear responses

It is common for the consequences of multiple stressors to be unpredictable based on knowledge of individual effects (Townsend et al. 2008). We combined wastewater and land‐use pressures, and found that agricultural land uses may ‘mask’ the detection of wastewater effects using Saprobic Index and Total Community Change (NMDS‐derived). Such antagonistic interactions are common amongst stressors in freshwaters, potentially reflecting the greater environmental variability of stream ecosystems that may help facilitate acclimation and co‐adaptation to multiple stressors (Jackson et al. 2016). However, we also found that wastewater impacts on the SPEAR Index were mostly additive to upstream pressures, reflecting the amount of wastewater entering the receiving stream.

Stress‐induced community sensitivity is predicted to occur where negative species co‐tolerances cause additive or synergistic responses in response to additional stressors (Vinebrooke et al. 2004). For example, Bunzel et al. (2013) showed stronger negative effects of upstream wastewater inputs on SPEAR taxa in more physically degraded streams, suggesting a synergistic interaction between pollution and habitat degradation. Rasmussen et al. (2012) similarly saw an increase in apparent pesticide effects with increasing habitat degradation, a result they explained by the reduction or absence of sediment‐sensitive SPEAR taxa. These changes may have also reflected differences in landscape properties, with lower recovery potential (i.e., recolonization of SPEAR) taxa) in heavily degraded habitats (e.g., urban streams). Streams with forested sections upstream can allow recolonization, thus greatly increasing the number and abundances of SPEAR taxa in pesticide‐affected stream sections (Liess and Ohe 2005). Environmental context is clearly an important factor to consider, and there could be practical limits to detecting impacts of pollution using community‐based approaches (i.e., changes may be asymptotic with increasing upstream pressures due to the effective loss of sensitive taxa). This supports using responses at multiple levels of biological organization, including populations (e.g., demographic, phenotypic, and/or genetic change) and at the ecosystem‐level (e.g., detrital processing rates) to measure effects (Thompson et al. 2015).

We tested responses to wastewater inputs using linear regression methods, after predicting community impairment to decrease with greater upstream pressures (e.g., agricultural land uses) or increase with the magnitude of wastewater disturbance. However, nonlinear responses could also be expected if crossing thresholds that lead to alternative stable states, characterized by altered community composition (Scheffer et al. 1993). Threshold responses in stream communities due to the influence of anthropogenic stressors may result in regime shifts between ‘sensitive’ and ‘resistant’ communities (Burdon et al. 2013; Taylor et al. 2014). Thus, alternative hypotheses for our study could be that greater upstream pressures might increase the sensitivity of upstream communities to additional environmental stress (i.e., stress‐induced community sensitivity), and/or adding wastewater inputs might move the system to a new ‘domain of attraction’ (Grimm and Wissel 1997; Vinebrooke et al. 2004). This could be by reducing populations of sensitive taxa below a ‘critical’ level or by exceeding nutrient thresholds affecting benthic algae (Ovaskainen and Hanski 2003; Taylor et al. 2014). In our study, the change in the relative abundance of oligochaetes may actually be a nonlinear relationship (Fig. 5), where increasing upstream degradation moves stream ecosystems to an alternative stable state (e.g., stress‐induced community tolerance) characterized by weaker responses to wastewater‐induced organic pollution.

### Recommendations for management

Changes in natural flow regimes due to urbanization, water abstraction, and climate change, may increase the impacts of pollutants through diminished flows, particularly in headwaters where stream discharges are low compared to edge habitat (Dodds and Oakes 2008). In our study, much of the negative non‐context dependent effects of wastewater were apparently driven by the magnitude of wastewater dilution. This indicates that (1) the amount of wastewater (i.e., that discharged relative to the dilution capacity of the receiving environment) should continue to be a management priority and (2) wastewater composition is an important environmental issue requiring advanced treatment technologies (Eggen et al. 2014).

There is a strong need for resource managers to plan for future contingencies in multi‐pressure catchments. Temporal variability in concentrations of “down‐the‐drain” chemicals in surface waters is strongly driven by flow variability (Keller et al. 2014), which may become particularly important with increased variation in precipitation due to climate change (Englert et al. 2013). For instance, in Switzerland rainfall is predicted to decrease by 18–27% in summer 2060–2090 relative to that in 1980–2009 (Vittoz et al. 2013). Moreover, the frequency and severity of extreme weather events is expected to increase, leading to longer droughts and more intense rainfall events (IPCC 2013). These changes, combined with the predicted warmer temperatures (Vittoz et al. 2013), may further contribute to stress imposed by pollutants associated with intensive agriculture and inputs of wastewater (Verberk et al. 2016).

Thus, reducing pollutant loads may have to be done jointly with rehabilitating stream habitats to enhance resilience for the impending problems posed by climate change (Palmer et al. 2009). For example, planting deciduous riparian trees along temperate streams as an adaptation to climate change can reduce temperatures by 2–3°C (Kristensen et al. 2013), and increase basal resources and macroinvertebrate biomass (Thomas et al. 2016). Importantly, the results of our study suggests that environmental contingency also needs to be accounted for when rehabilitating degraded stream reaches, because catchment properties may affect the ecological responses of the restoration, irrespective of the remediation effort (Kowalik and Ormerod 2006; Sundermann et al. 2011).

### Concluding remarks

Overall, our findings highlight the need to consider differing impacts across spatially heterogeneous landscapes, where catchment‐scale pressures influence the structure of stream invertebrate communities and their responses to local disturbances. Conversely, although we found that catchment land uses (i.e., arable cropping) were associated with reductions in populations of sensitive invertebrates, this did not necessarily make communities more or less sensitive to toxicants (e.g., pesticides) discharged from WWTPs. Wastewater influences (i.e., toxicants) may scale with dilution factors, further illustrating the continuing need to manage the quality and quantity of treated effluents from WWTPs. Thus, catchment‐wide pollution from diffuse (e.g., agriculture) and point‐sources (e.g., municipal wastewater) continue to be major ecological problems requiring multiple strategies to reduce inputs into receiving environments.

## Conflict of Interest

None declared.

## Supporting information


**Appendix A.** Study sites.
**Appendix B.** Detailed method descriptions.
**Appendix C.** Results complementing the main text
