# Supplementary material for: Environmental context and magnitude of disturbance influence trait‐mediated community responses to wastewater in streams
Source: Ecol Evol. 2016 May 12;6(12):3923–39. doi: 10.1002/ece3.2165 (PMC4972221; doi:10.1002/ece3.2165)
Supplement: Supplementary file 1 — Appendix A. Study sites. Appendix B. Detailed method descriptions. Appendix C. Results complementing the main text [file ECE3-6-3923-s001.docx]

Supporting information for:

**Environmental context and disturbance influence trait-mediated community responses to wastewater pollution in streams**

F. J. Burdon^1^, [Francis.Burdon@eawag.ch](mailto:Francis.Burdon@eawag.ch); M. Reyes^1^, [Marta.Reyes@eawag.ch](mailto:Marta.Reyes@eawag.ch); A. C. Alder^1^, [Alfredo.Alder@eawag.ch](mailto:Alfredo.Alder@eawag.ch); A. Joss^1^, [Adriano.Joss@eawag.ch](mailto:Adriano.Joss@eawag.ch); C. Ort^1^, [Christoph.Ort@eawag.ch](mailto:Christoph.Ort@eawag.ch); K. Räsänen^1^, [Katja.Rasanen@eawag.ch](mailto:Katja.Rasanen@eawag.ch); J. Jokela^1,2^, [Jukka.Jokela@eawag.ch](mailto:Jukka.Jokela@eawag.ch); R. I. L. Eggen^1,2^, [Rik.Eggen@eawag.ch](mailto:Rik.Eggen@eawag.ch); C. Stamm^1^ [Christian.Stamm@eawag.ch](mailto:Christian.Stamm@eawag.ch)

1. Eawag, Swiss Federal Institute of Aquatic Science and Technology, Dübendorf, Switzerland
2. ETH-Zurich, Swiss Federal Institute of Technology, Zurich, Switzerland

Corresponding author: Dr. Francis J. Burdon

Aquatic Ecology - Eawag

P.O. Box 611, 8600 Dübendorf

Switzerland

francis.burdon@eawag.ch

Phone +41 58 765 6713

Fax +41 58 765 5028

**Introduction**

The Supporting Information are structured in three parts:

1. Detailed information regarding the study sites and the sampling scheme (Appendix A)
2. Detailed descriptions of methods including field sampling, metrics for community analysis, and statistical procedures (Appendix B).
3. Complementary results that supplement the information provided in the main text (Appendix C).

**Appendix A: Study sites**

**A.1 Site selection and study design**

The study sites were selected across the the Swiss Plateau, Pre-Alps, and Jura mountains bioregions (Fig. A1). Their catchments vary considerably in size, land use, and biogeographical setting (Table A1).

At each of the 12 study sites, we determined one downstream sampling location (D), and two upstream sampling locations (U1, U2). The distance from the WWTP for reaching a homogeneous conductivity profile at low flow conditions was defined as the mixing distance d_mix_ (Fig. A2). This was determined by measuring specific conductivity (ProfiLine Cond 3110, WTW GmbH, Weilheim, Germany) on sequential transects across the stream moving downstream of the WWTP discharge. Two upstream locations (U1, U2) were selected as controls. Location U1 was chosen as closely to the WWTPs discharge as possible, and U2 approximately equidistant to that between U1 and D (Fig. A2). Exact locations were adjusted to achieve the best possible similarity in habitat (e.g., stream geomorphology).

Table A1 Characterization of the study sites and their catchments for the twelve Swiss streams sampled between March 2013 and February 2014. Region: J: Jura, Pl: Swiss Plateau, PA: Pre-alps; according to the Swiss Modular Stepwise Procedure ([Schaffner, Pfaundler & Göggel 2013](#_ENREF_24)). *Q*_347_ is the discharge which is reached or exceeded 347 days per year averaged over ten years (equivalent to 95% of the time). The WW dilution factor was calculated as *Q_347_* / *Q_WW_*_,_ where *Q_WW_* is the annual mean discharge of wastewater (l/s). Discharge data was obtained from the GEWISS water information system ([www.bafu.admin.ch/wasser/13462/13496/15866](http://www.bafu.admin.ch/wasser/13462/13496/15866)). Landuse data (“Arealstatistik 2009”) was obtained from Swiss landuse statistics collected 2004-2009 ([www.landuse-stat.admin.ch](http://www.landuse-stat.admin.ch)). Catchment areas were estimated from the dataset “GAB-EZGG-CH” ([www.bafu.admin.ch/wasser/13462/13496/15009](http://www.bafu.admin.ch/wasser/13462/13496/15009)).

| Site | Code | Region | Elevation  (m a.s.l) | Discharge (Q_347_ l/s) | WW dilution factor (Q_347_) | Forest (%) | Pasture (%) | Cropping (%) | Orchard (%) | Urban (%) | Catchment area (ha) |
| --- | --- | --- | --- | --- | --- | --- | --- | --- | --- | --- | --- |
| Buttisholz | BUT | Pl | 548 | 23 | 1.2 | 15 | 34 | 46 | 0.1 | 8 | 1674 |
| Colombier | COL | Pl | 492 | 17 | 4.2 | 9 | 5 | 45 | 1.6 | 8 | 763 |
| Dürnten | DUR | Pl | 495 | 75 | 1.0 | 11 | 43 | 22 | 0.2 | 20 | 1087 |
| Herisau | HER | PA | 698 | 127 | 0.7 | 29 | 52 | 0 | 0.0 | 12 | 3990 |
| Hochdorf | HOC | Pl | 470 | 172 | 1.0 | 14 | 35 | 38 | 1.1 | 10 | 4946 |
| Hornussen | HOR | J | 370 | 20 | 1.0 | 39 | 25 | 27 | 1.3 | 9 | 2928 |
| Kernenried | KER | Pl | 505 | 362 | 1.5 | 34 | 10 | 37 | 0.0 | 14 | 8226 |
| Messen | MES | Pl | 470 | 121 | 3.4 | 24 | 14 | 52 | 0.1 | 7 | 5071 |
| Niederdorf | NIE | J | 459 | 114 | 1.7 | 46 | 28 | 10 | 0.2 | 10 | 1889 |
| Romont | ROM | Pl | 688 | 209 | 1.8 | 16 | 60 | 18 | 0.1 | 8 | 9865 |
| Rothenthurm | ROT | PA | 912 | 37 | 3.4 | 45 | 30 | 0 | 0.0 | 4 | 2280 |
| Sévéry | SEV | Pl | 597 | 16 | 2.6 | 31 | 11 | 48 | 0.0 | 5 | 1347 |


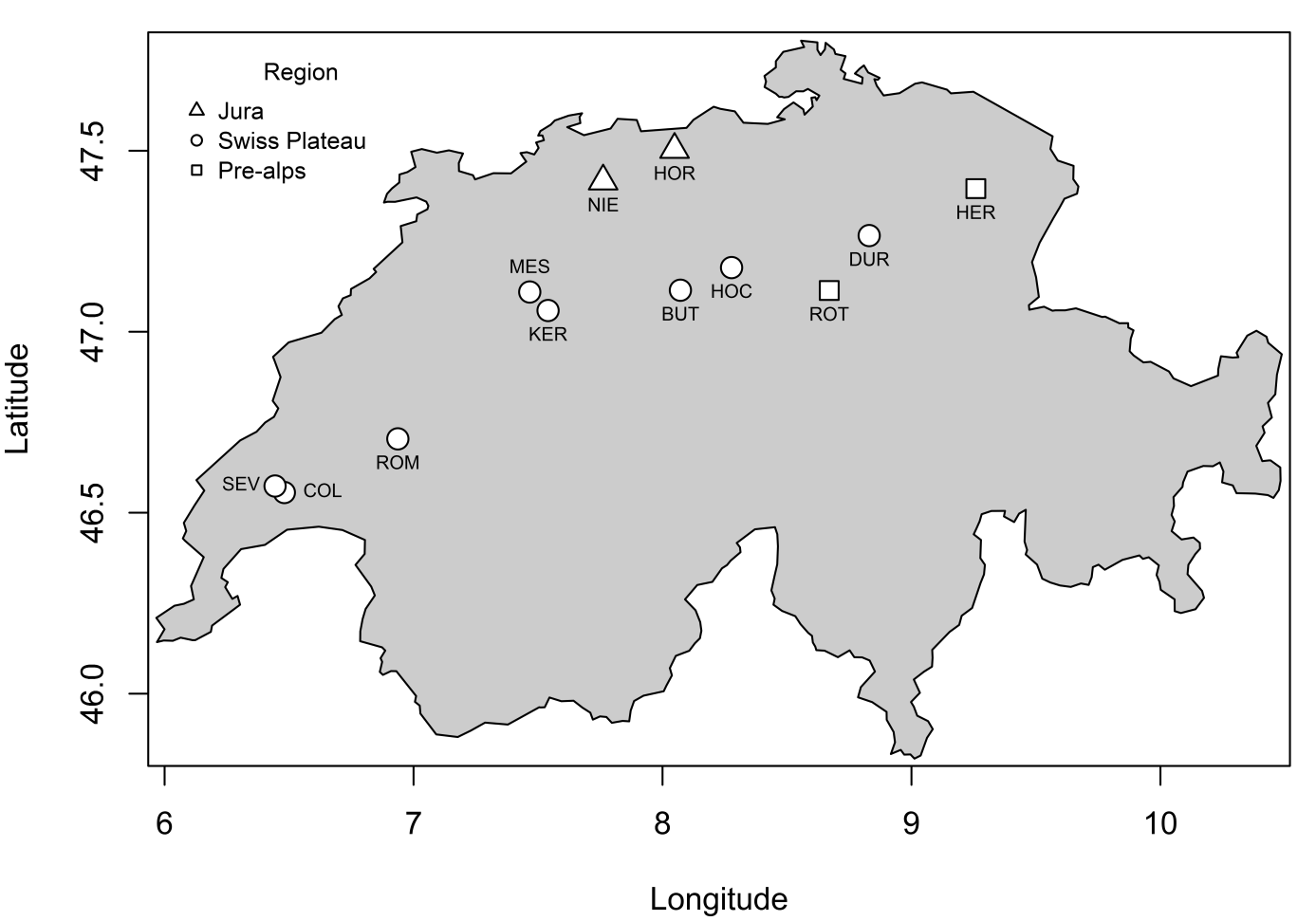


**Figure A1** Map of Switzerland showing the location of the study sites.


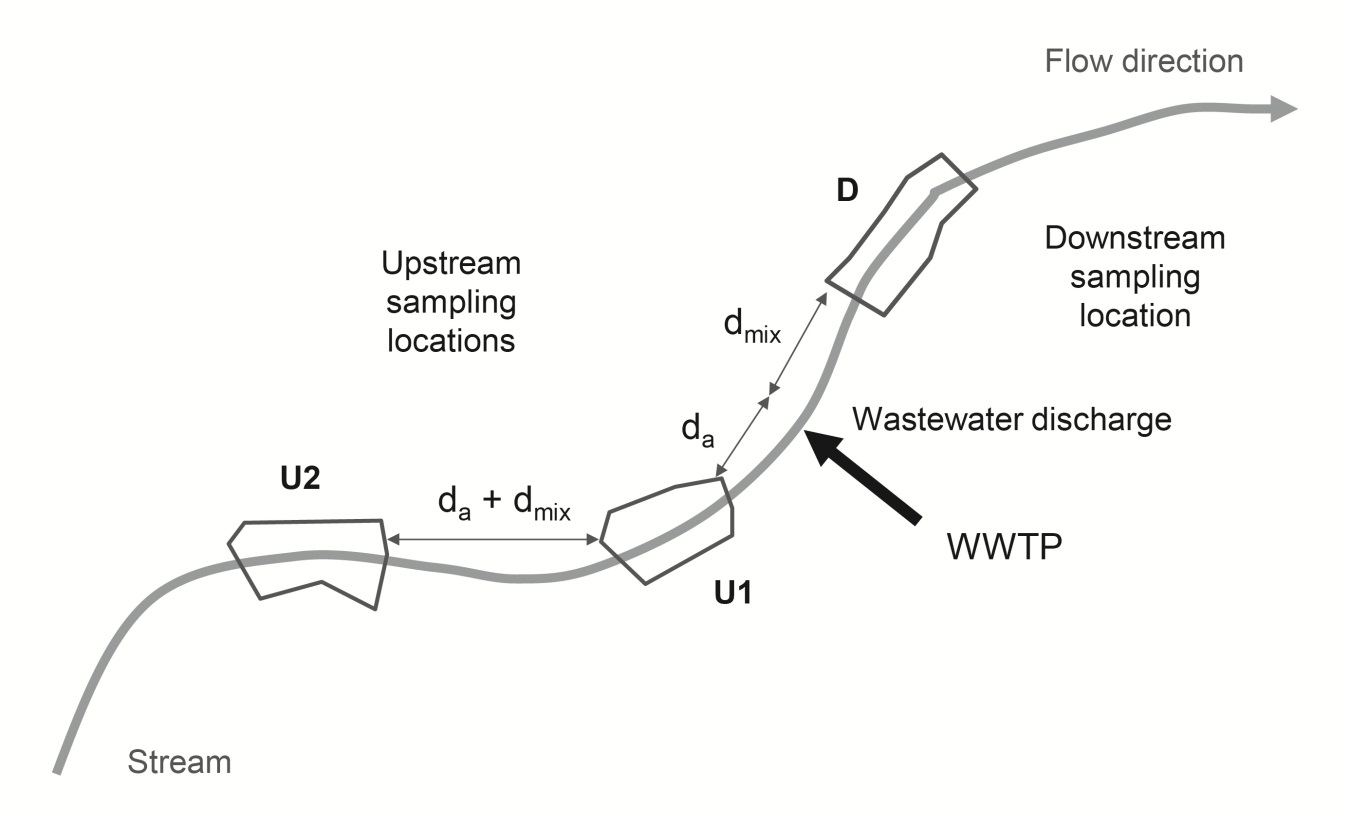


**Figure A2** Sampling scheme at each study site showing the location of downstream (D) and upstream (U1 and U2) sampling points. *d_mix_*, distance downstream for homogenous mixing of wastewater with surface water; *d_a_*, distance between the wastewater discharge and first upstream sampling point (U1).

**Appendix B: Detailed method descriptions**

**B.1 Field methods**

***B.1.1 Estimation of benthic suspendible sediment***

The amount of benthic suspendible sediment (SS_benthic_; kg/m) was estimated using the ‘Quorer method’ ([Quinn *et al.* 1997](#_ENREF_22)). SS_benthic_ is the sum of suspendible inorganic sediment (SIS_benthic_) and suspendible organic sediment (SOS_benthic_), as defined by [Clapcott *et al.* (2011](#_ENREF_8)). First, grab water samples were taken from the water column to determine the background level of total suspended sediments (TSS_river_). Then the benthic substrate in an open drum (20 cm diameter, 0.03 m^2^) at eight random locations within each sampling reach was vigorously disturbed using a steel rod (duration of 30 s) to collect replicate samples of total suspended sediment (TSS_benthic_; i.e., a 250-mL water column sample taken from inside the drum). TSS_benthic_ is the sum of the background level (TSS_river_) and the benthic suspendible sediments (SS_benthic_). Samples were placed in a cooling box on ice for transport before storage at 4°C in the laboratory until analysis. They were processed within 48 hours of collection.

To estimate the organic and inorganic fractions of the suspendible sediment, a subsample (50 mL) of the water sample was then ﬁltered through a pre-ashed, pre-weighed ﬁlter (GF/C, 47 mm diameter), before being dried for 48 hours at 48° C, weighed, ashed for 4 hours at 400° C, and reweighed. The proportion of organic sediment was calculated by dividing the difference between the dried and ashed filter by the pre-ashed dry mass of the sample minus the mass of the filter. The estimates of benthic suspendible sediment were corrected for the background levels of suspended particles TSS_river_ (Eq. 1):

| ${SS}_{benthic}={TSS}_{benthic}-{TSS}_{river}$ | (1) |
| --- | --- |

**B.2 Description of invertebrate communities**

To describe invertebrate community responses above and below wastewater inputs in the main text, we calculated four diversity indices and two trait-based indicators of stream health using the invertebrate community data at each sampling location. The diversity indices were all obtained using the “vegan” package in R (Oksanen et al. 2013).

**B.2.1 Diversity indices**

*B.2.1.1 Taxa and rarefied richness*

Taxa richness was determined from counts of invertebrate taxa. To make them comparable across sites and locations, rarefied taxa richness was calculated using the “vegan” function ‘rarefy’ which is based on Hurlbert's ([1971](#_ENREF_11)) formulation (Eq.2):

| $E(S_{n})=\sum_{i} \left[ 1-\frac{\left( \frac{N-N_{i}}{n} \right)}{\left( \frac{N}{n} \right)} \right]$ | (2) |
| --- | --- |

Where *E*(*S_n_*) is the expected number of species in a sample of *n* individuals selected at random from a collection containing *N* individuals, *S* species, and *N_i_* individuals in the *i*th species. All rarefied taxa richness values were derived for the minimum number of individuals observed at a study reach (*N* = 779).

*B.2.1.2 Taxa evenness*

The evenness of the macroinvertebrate communities per location was represented by Pielou’s evenness index (J), which is described as:

| $J=\frac{H}{\ln S}$ | (3) |
| --- | --- |

where *H* represents the Shannon-Wiener index for a sampling location (see below; Eq. 4), and *S* is the total number of taxa at the same location ([Pielou 1975](#_ENREF_20)).

The Shannon-Wiener Diversity Index ([Shannon 1948](#_ENREF_27)) is a measure of information entropy, and is described as *H*:

| $H=-\sum_{i=1}^{R} p_{i}\ln p_{i}$ | (4) |
| --- | --- |

In this context, *p_i_* is the proportion of individuals belonging to the *i*th taxon in the community at a single location.

*B.2.1.3 Fisher's alpha*

Fisher’s alpha is an implicit function of Fisher’s log-series distribution parameter and of total community abundance ([Magurran 2004](#_ENREF_16)). It is often used as a synthetic diversity index, but as it tends to converge to the number of singletons in a community, it is an indicator of the number of rare species ([Magurran 2004](#_ENREF_16)). The dimensionless *α* parameter can be estimated from Fisher’s log-series ([Fisher, Corbet & Williams 1943](#_ENREF_10)), where the expected number of species *f* with *n* observed individuals is:

| $E(S_{f})=\frac{\alpha x^{n}}{n}$ | (5) |
| --- | --- |

The estimation of *α* follows [Kempton and Taylor (1974](#_ENREF_12)). The parameter *x* is taken as a nuisance parameter which is not estimated separately ([Oksanen *et al.* 2013](#_ENREF_17)), but instead described as:

| $x=\frac{n}{\left( n+ \alpha\right)}$ | (6) |
| --- | --- |

**B.2.2 Trait-based community descriptors**

In addition to the diversity indices above, we calculated two indices of stream health using a trait-based community approach.

*B.2.2.1 Saprobic Index*

Despite the extensive technological improvements in wastewater treatment, modern WWTPs can still be an important source of oxygen-depleting organic pollution ([Bunzel, Kattwinkel & Liess 2013](#_ENREF_6)). Thus, we also used the invertebrate community data to calculate an index reflecting the saprobic condition of our sites. This was based on the German Saprobity Index, which is the core metric of organic pollution within the official German Water Framework Directive assessment system for macroinvertebrates ([Bunzel, Kattwinkel & Liess 2013](#_ENREF_6)).

German saprobic trait values for relevant taxa were obtained from: [www.freshwaterecology.info](http://www.freshwaterecology.info). This online resource is a taxa and autecology database for freshwater organisms (Version 5.0, Date accessed: 26.03.15; for more information see Schmidt-Kloiber & Hering 2015). Where relevant trait values were not available for taxa using German scores, alternative values from Austria and Slovakia were used. We used mean values for taxa where there were multiple genera within each group. Saprobic Index scores for individual taxa are listed in Table C7, Table Appendix C. The Saprobic Index (SI), a number between 1 and 4, is the “weighted mean” of all individual indices calculated as:

| $SI={\sum_{i=1}^{n} \left( S_{i}\times a_{i} \right)}/{\sum_{i=1}^{n} a_{i}}$ | (7) |
| --- | --- |

where for any given taxa *i* the product of abundance *a_i_* and saprobic trait score *S_i_* expresses the saprobic value for that taxon. Higher SI values indicate shifts in the macroinvertebrate community towards species that are more tolerant of low oxygen conditions ([Bunzel, Kattwinkel & Liess 2013](#_ENREF_6)).

The Saprobic index was calculated in Excel (Microsoft Excel 2010, Version 14).

*B.2.2.2 SPEAR Index*

The Species At Risk (SPEAR) index used is designed to detect and quantify the effects of pesticides (e.g., insecticide toxicity) on macroinvertebrate communities ([Schäfer *et al.* 2007](#_ENREF_23); [Schriever *et al.* 2007](#_ENREF_26)). This multiple trait-based approach links pesticide stress and community composition by utilizing traits that reflect the ecological requirements of the invertebrates and pesticide effects (Liess & Ohe 2005). The traits involved are 1) the physiological sensitivity to organic toxicants, 2) generation time, 3) presence of aquatic life stages during exposure, and 4) recovery potential. The SPEAR pesticides index is calculated as the relative abundance of sensitive taxa "at risk" to be affected by pesticides:

| $\mathrm{SPEAR}_{\mathrm{pesticides}}=\frac{\sum_{i=1}^{n} \log\left( x_{i}+1 \right)y}{\sum_{i=1}^{n} log(x_{i}+1)}$ | (8) |
| --- | --- |

where *n* is the number of taxa, *x_i_* is the abundance of taxon *i,* and *y* is a binary variable (1 if taxon *i* is classified as SPEAR, 0 if not). Calculations were performed to the lowest practicable taxonomic levels for abundances; research suggests that the explanatory power of the family-level SPEAR pesticides is not significantly lower than the species-level index ([Beketov *et al.* 2009](#_ENREF_3)). Two very rare taxa (Cnidaria, Stratiomyidae) not recognised in the SPEAR traits database were excluded from calculations. Taxa that were classified as SPEAR are listed in Table C7, Appendix C.

The SPEAR pesticides index was calculated using the R package ‘rSpear’ ([Szoecs 2013](#_ENREF_29)).

**B.2.3 Additional community descriptors**

We also calculated seven additional taxonomic and trait-based community descriptors not reported in the main text. Mean values (± 1SD) at different sampling locations and results from mixed–models analysis can be seen in Table C6, Appendix C.

*B.2.3.1 Abundances*

The changes in the raw abundances of all invertebrates collected in kick-net samples are reported in Table C6, Appendix C. Abundances minus the oligochaete worms are also included.

*B.2.3.2 EPT richness and relative abundance*

The number of Ephemeroptera, Plecoptera, and Trichoptera (EPT) insect taxa and the proportion of EPT (% EPT by abundance) are two commonly used measures of community composition and stream health ([Burdon, McIntosh & Harding 2013](#_ENREF_7)). Relative abundances of EPT minus the oligochaete worms are also included.

*B.2.3.3 Berger-Parker Dominance Index*

The Berger–Parker index ([Berger & Parker 1970](#_ENREF_4)) equals the maximum *p_i_* value in the invertebrate data at each sampling location (i.e., the proportional abundance of the most abundant taxon).

*B.2.3.4 IBCH*

The IBCH index is the French IBGN (*Indice Biologique Global Normalisé*) adapted for Switzerland ([Stucki 2010](#_ENREF_28)). It uses the presence of predefined indicator taxa in relation to total macroinvertebrate taxa richness to derive a score of stream health. Indicator taxa are grouped according to their ecological requirements, with lower scoring taxa generally more pollution tolerant. Thus, a low IBCH score (e.g.,< 5) is used to indicate a habitat of ‘poor’ ecological status, whereas a high IBCH score (e.g., > 17) represents ‘very good’ ecological status ([Stucki 2010](#_ENREF_28)).

**B.3 Detailed descriptions of data analysis methods**

**B.3.1 Effect sizes using Cohen’s *d***

Mean standardized effect sizes ([Cohen 1992](#_ENREF_9)) were calculated between D and upstream controls (U1 and U2) to show the relative magnitude of changes shown by the different indices used (Table 1, Main text). Cohen’s *d* quantitatively shows the strength of the change in different invertebrate community metrics downstream (D) compared to upstream locations (U1, U2) after accounting for pooled variation among sampling points. Cohen's d is defined as the difference between two means divided by a standard deviation for the data:

| $d=\frac{\bar{x}_{1}-\bar{x}_{2}}{s}$ | (9) |
| --- | --- |

**B.3.2 Ordinations of community data**

*B.3.2.1 Unconstrained ordination (non-metric multi-dimensional scaling)*

To compare changes to community composition above and below the wastewater inputs, we used non-metric multi-dimensional scaling (NMDS) to summarize macroinvertebrate data in relation to the study reaches (downstream versus upstream controls) in the twelve streams. We repeated this analysis on invertebrate community data omitting the Oligochaeta, a hyper-abundant taxon group at downstream sites (Fig. C3A, Appendix C).

NMDS is an unconstrained ordination technique that maps samples in reduced dimensional space using only the rank order of dissimilarity values among samples ([Quinn & Keough 2002](#_ENREF_21)). Relative abundances of taxa at each location (abundance of taxa *i* expressed as a fraction of sum of abundances of all taxa at the respective location) were Hellinger-transformed. This offers the advantage of not strongly weighting rare taxa in the analyses ([Legendre & Gallagher 2001](#_ENREF_13)). The NMDS solution was determined in two, three, and four dimensions using multiple random starts to maximize the likelihood of reaching a global minimum stress value, but only the two-dimensional solution was retained because stress did not decline appreciably with additional dimensions.

To determine differences in community composition between sampling locations, we first conducted a permutational multivariate analysis of variance (PERMANOVA) on the relative abundance data from sampling locations D, U1, and U2 using the Euclidean-distance metric following Hellinger transformation. We followed this test with two individual PERMANOVAs testing the pairwise differences between D and U1, and U1 and U2 at eleven study streams. One study site (Kernenried) was omitted from these analyses as it appeared as a clear outlier in the initial NMDS plot (Fig. C2, Appendix C) owing to exceptionally high proportions of gammarid amphipods at all three sampling locations. This did not materially affect the results of the tests, but did improve their explanatory power. The pairwise comparisons were Bonferroni-corrected for multiple comparisons to avoid a Type I error.

*B.3.2.2 Constrained ordination (partial redundancy analysis)*

We used a total of four partial redundancy analysis (pRDA) models to test the association of landuse, water quality and instream habitat with invertebrate community composition after conditioning out the effects of spatial location (i.e., spatial structuring leading to autocorrelation of communities). Two pRDA models tested environmental determinants and spatial influences on upstream communities only (U1,U2), and two pRDA models tested similar environmental and spatial influences on communities from all three sampling locations (D,U1,U2).

The first two pRDA models tested these environmental determinants and spatial influences at the upstream sampling locations (U1,U2). The first model indicated the correlation of two trait-based invertebrate indices (Saprobic and SPEAR pesticides) and quantified the amount of variability in community composition that could be attributed catchment landuses after accounting for the confounding effect of spatial location (Fig. C1A, Appendix C). The second model used local environmental factors (water quality and instream habitat charcteristics) and catchment landuses after accounting for the confounding effect of spatial location (e.g., Fig.C1B,C, Appendix C). The taxa scores from the first three axes (RD1-3) for upstream sampling location U1 from the partial redundancy analysis of upstream invertebrate communities (Fig. C1C) were used as an indicator of community composition (associated with saprobic condition, see Fig. 4B, Main text) in our regression analyses.

The two ‘Full’ pRDA models tested the influences of physicochemical parameters on community composition at all three sampling locations (D, U1, U2). The first model quantified the amount of variability in community composition that could be attributed to water quality and habitat parameters whilst accounting for the confounding effect of stream (i.e., upstream controls and downstream sites were not statistically independent) and spatial location (i.e., Fig. 3B, Main text). The second model using invertebrate community data minus the Oligochaeta (a taxon group that were hyperabundant at downstream sites, and thus highly influential on the original unconstrained ordination shown in Fig. 3A, Main text). The results of pRDA (minus Oligochaeta) are shown in Fig. C3B, Appendix C.

We used Hellinger-transformed relative abundance data to create the matrices of distances between sites reflecting the community composition (see above). The local environment was described water chemistry and instream habitat data. Water quality variables initially composed of twenty measured parameters using average monthly data, and the habitat data comprised of the subjective categories used for visual assessment (Table 1, Main text), and physical measurements of total suspendible sediment (g/m^2^) and the proportion of organics (% organic sediment), mean wetted-channel widths (m), water depths (m), and flow velocities (m/s). Shapiro tests were used to identify non-normal variables which were log-transformed prior to further analysis, and proportion data was arscine square-root-transformed. All variables were centred on the column means and standardised (i.e., divided by the standard deviation) prior to analysis.

Spatial location was represented by a matrix generated using Principal Coordinates of Neighbors Matrices (PCNM) analysis. PCNM is a method for detecting and quantifying spatial patterns over a range of difference scales. Site locations were described using the Swiss coordinate system using the equivalents of latitude and longitude in this Cartesian system (x, y). Using the x and y coordinates; matrices of PCNM variables were created using the “pcnm” command in R. The default truncation distance was used; this is the longest distance to keep data connected ([Borcard & Legendre 2002](#_ENREF_5)).

To avoid over-parameterisation of the pRDA models using spatial, catchment, and local environmental data, Pearson product-moment correlation tests were used to discard parameters that were strongly collinear (*r* > 0.6), and forward-selection of variables using permutation tests was used to remove factors that did not significant explain variation in invertebrate community composition.

The variation partitioning methods simultaneously quantified the amount of variability in community composition that could be attributed to water quality and habitat parameters independently and jointly (i.e., covariance) whilst accounting for the confounding effect of stream (i.e., upstream controls and downstream sites were not statistically independent) and spatial location. Residuals that were not explained by water quality, habitat, stream and spatial location were considered to be the unexplained variation. Proportions of the total variation attributed to each component were based on the adjusted (unbiased) fractions, which consider in each analysis the total number of predictors and the sample size. The significance of each fraction was tested by permutation tests using 1000 randomizations ([Peres-Neto, Jackson & Somers 2003](#_ENREF_19)).

pRDA analyses were performed in R using the package “vegan” ([Oksanen *et al.* 2013](#_ENREF_17)).

**B.3.3 Multivariate analysis of community change**

*B.3.3.1 Multiple regression models*

The main objective of our research was to better understand how much community responses are determined by environmental context (e.g., catchment influences on water quality and gamma diversity) as opposed to the magnitude of disturbance (i.e., pollution levels reflecting wastewater quantity and composition).

We used multiple-regression models to account for the influences of wastewater quantity (i.e., dilution factors), WW composition (i.e., wastewater quality parameters), instream habitat change (between upstream and downstream sites), and upstream (i.e., reference) community composition as a proximate indicator of catchment ecological integrity.

*B.3.3.2 Habitat change metric used in multiple regression models*

Principal components analysis (PCA) was also used to describe the change in physical habitat between upstream and downstream sites. This habitat data comprised of the same subjective categories and physical measurements described previously (Table 1, Main text). So that habitat changes were not confounded by wastewater impacts, two habitat parameters (% of organic suspendible sediment and % cover of submerged macrophytes) associated with altered invertebrate community composition in response to wastewater discharges (Fig. 3B, Main text) were excluded from this analysis. The distance in three-dimensions (i.e., PCA axes 1, 2, and 3) between D and the upstream sites U1 (Eq.10), after correcting for the ‘null’ change in habitat between sites U1 and U2 (Eq.11), was used as a measure of habitat change (Eq.12):

| ${Habitat \Delta}_{i}=\sqrt{{\left( {PC1}_{D}-{PC1}_{U1} \right)_{i}}^{2}+{\left( {PC2}_{D}-{PC2}_{U1} \right)_{i}}^{2}+{\left( {PC3}_{D}-{PC3}_{U1} \right)_{i}}^{2}}$ | (10) |
| --- | --- |
| ${Null habitat \Delta}_{i}=\sqrt{{\left( {PC1}_{U1}-{PC1}_{U2} \right)_{i}}^{2}+{\left( {PC2}_{U1}-{PC2}_{U2} \right)_{i}}^{2}+{\left( {PC3}_{U1}-{PC3}_{U2} \right)_{i}}^{2}}$ | (11) |
| ${Corrected habitat \Delta}_{i} ={Habitat \Delta}_{i}-{Null habitat \Delta}_{i}$ | (12) |

where *i* is the study stream, *U1* and *U2* are the upstream sites, and *D* is the downstream site. The PCA in three-dimensions explained 45% of the variation in instream habitat amongst sites and sampling points.

*B.3.3.3 Hierachical partitioning and partial correlations*

In all the multiple-regression models, correlations with response variables were tested using hierarchical partitioning to evaluate the independent effects of the predictor variables. This approach used R^2^ values to determine the proportion of variance explained independently and jointly by variables, where all possible models in a multiple regression setting are considered ([Mac Nally 2000](#_ENREF_15)). The statistical significance of these variables was based on the upper 95% confidence limit (Z-score > 1.65; Mac Nally 2000); analyses were conducted using the “hier.part” package in R ([Walsh & Mac Nally 2007](#_ENREF_30)). Additionally, partial correlations were performed when predictor variables were significant to provide an indication of the direction of the relationship with the response variable. Pearson’s product-moment partial correlations were conducted using the ‘ppcor’ package in R .

**B.3.4 Beta-diversity partitioning**

To disentangle the contribution of spatial turnover (species replacements) and nestedness (species losses) to beta-diversity patterns, we used the procedure described by Baselga (2010). This procedure uses additive partitioning of beta diversity to provide the two separate components of spatial turnover and nestedness that underlie the total amount of beta diversity ([Baselga 2010](#_ENREF_1)). Pairwise estimates of dissimilarity between sampling locations were obtained using taxa occupancy data. Pairwise dissimilarities of Sorenson’s, Simpson’s and the nestedness-resultant measures (i.e., indices) were calculated for each site using the ‘betapart’ package in R ([Baselga & Orme 2012](#_ENREF_2)). Differences in pairwise measures of dissimilarity between the downstream site (D-U1) and the upstream controls (U1-U2) were then tested using blocked ANOVAs (i.e., blocking for the influence of site). Results are reported in C4.3 and Fig. C4, Appendix C.

**Appendix C: Results complementing the main text**

In this section, we provide additional data on water quality, the two main drivers of community change, the influence of habitat quality, and the changes observed in the invertebrate communities.

**C.1 Water quality**

Due to the geology of the study region, which is dominated by calcareous material, the study streams showed high values for hardness and specific conductivity (median 2.8 mmol/l and 508 µS/cm at 20° C respectively at the upstream locations; see Table C1).

The water quality data indicated that varying levels of anthropogenic stress was exerted on the streams at the upstream locations (see Main text). Only 6 sites (for soluble reactive phosphorus) and 7 sites for P_total_ fell into the (very) good classes according to the Swiss assessment protocols ([Liechti 2010](#_ENREF_14)). The remaining sites were classified as being moderate to very bad for this parameter. Similarly, organic carbon levels were elevated in some stream: 3 sites (for TOC) and 5 sites (for DOC), respectively, missed the (very) good classes. Generally, however, the nitrogen levels (NH_4_^+^, NO_3_^-^, N_total_) at the upstream locations were better. All but one site revealed a (very) good chemical status with regard to these nitrogen parameters.

Inputs of wastewater changed water quality downstream markedly. Only pH showed a minor decrease at downstream sites (-0.15% median decrease). In contrast, nutrients showed the greatest increases, including ammonia (101% median increase), nitrite (122%), and dissolved phosphorus (139%).

Wastewater from all WWTPs was sampled twice (June 2013, January 2014). Summary statistics are provided in Table C2. To reduce the number of explanatory variables for subsequent analyses, the measurements taken at each WWTP were reduced to three components with a principal components analysis (PCA). The results are represented in Table C3. The first component (PC1) could be interpreted as representing the human influence in general, while PC2 and PC3 show indications of high phosphorous concentrations (i.e., poor P elimination) and high nitrite concentrations (i.e., poor nitrification) in the WWTPs, respectively.

**C.2 Comparing environmental context and magnitude of disturbance**

We used catchment land uses as the ‘ultimate’ representation of environmental context. Catchment land uses differed considerably in their intensity across study sites, with arable cropping approximately ranging from 0–52% and pasture 5–60% of total catchment area respectively. Combined, these land uses ranged from approximately 30–80%, in contrast to the 9–46 % for low intensity forested land cover. We used the dilution potential of wastewater as the main measure for the magnitude of disturbance. Wastewater dilution factors based off the *Q_347_* ranged from less than one (0.75; low dilution potential) to 4.23 (high dilution potential). Thus, the receiving streams had wastewater loads ranging from 19% to 57% of total discharge (*Q_347_*) below the outfall. This meant that similar to the environmental context (e.g., water quality), the disturbance exerted by the discharge of WW into the 12 study streams varied substantially among sites.

The coefficient of variation (CV) of ‘environmental context’ drivers compared favourably with that of the ‘magnitude of disturbance’ (i.e., wastewater dilution factors), meaning the gradients of these predictors were comparable in scope. We assumed the ultimate drivers of ‘environmental context’ influences were the proportions of agricultural land uses (cropping and pasture) in study catchments. The CV for cropping (0.65) and pasture (0.60) were comparable with that of wastewater dilution factors (0.59). When the two land use categories were combined, their CV (0.27) was similar to the CV for the log-transformed wastewater dilution factors (0.36). Although these statistical properties do not necessarily reflect actual impacts, the range of values for both environmental context and magnitude of disturbance drivers suggested that strong to moderate effects could be expected for both factors (see also Results, Main Text).

**Table C1** Summary statistics of site-averaged water chemistry variables measured monthly for twelve Swiss streams sampled between March 2013 and February 2014. D, sampling point downstream of wastewater discharge; U1, 1^st^ upstream sampling point; U2, upstream 2; TN, total nitrogen; SRP, soluble reactive phosphorus; TP, total phosphorus; TOC, total organic carbon; DOC, dissolved organic carbon; SS, suspended solids.

|  |  |  | Sampling point | | | | | | | | | | | | | |
| --- | --- | --- | --- | --- | --- | --- | --- | --- | --- | --- | --- | --- | --- | --- | --- | --- |
|  |  |  | D | | | |  | U1 | | | |  | U2 | | | |
| Variable | Unit |  | Mean | Median | Min. | Max. |  | Mean | Median | Min. | Max. |  | Mean | Median | Min. | Max. |
| Conductivity | µS/cm 20° C |  | 562 | 562 | 352 | 714 |  | 515 | 508 | 342 | 679 |  | 516 | 506 | 340 | 685 |
| pH |  |  | 8.2 | 8.2 | 8.1 | 8.3 |  | 8.2 | 8.2 | 8.1 | 8.3 |  | 8.2 | 8.2 | 8.1 | 8.3 |
| Alkalinity | mmol/L |  | 5.1 | 5.2 | 3.8 | 5.7 |  | 5.1 | 5.3 | 3.8 | 5.7 |  | 5.1 | 5.3 | 3.8 | 5.7 |
| Hardness | mmol/L |  | 2.9 | 2.9 | 2.0 | 4.1 |  | 2.9 | 2.9 | 2.0 | 4.1 |  | 2.9 | 2.8 | 1.9 | 4.1 |
| Na^+^ | mg/L |  | 19.6 | 15.3 | 6.6 | 45.5 |  | 10.4 | 8.7 | 5.1 | 31.2 |  | 11.4 | 8.8 | 5.0 | 43.4 |
| K^+^ | mg/L |  | 3.6 | 3.5 | 1.8 | 5.8 |  | 2.4 | 2.4 | 1.3 | 3.4 |  | 2.5 | 2.3 | 1.4 | 5.2 |
| Ca^2+^ | mg/L |  | 97.1 | 97.1 | 64.1 | 131.1 |  | 96.6 | 98.0 | 64.4 | 133.4 |  | 96.8 | 99.3 | 63.3 | 134.2 |
| Mg^2+^ | mg/L |  | 11.7 | 10.9 | 7.6 | 19.1 |  | 11.9 | 10.7 | 7.6 | 19.5 |  | 11.7 | 10.6 | 6.2 | 19.7 |
| NH_4_^+^ | µg/L |  | 118.4 | 54.4 | 12.6 | 544.7 |  | 37.1 | 33.2 | 8.0 | 108.3 |  | 31.5 | 31.7 | 7.4 | 70.9 |
| NO_2_^-^ | µg/L |  | 26.6 | 25.2 | 7.1 | 46.9 |  | 13.4 | 12.8 | 2.3 | 30.1 |  | 13.2 | 12.9 | 1.8 | 32.7 |
| NO_3_^-^ | mg/L |  | 4.6 | 4.4 | 1.0 | 7.4 |  | 3.1 | 3.2 | 0.8 | 6.4 |  | 3.1 | 3.1 | 0.8 | 6.3 |
| TN | mg/L |  | 6.5 | 7.0 | 1.2 | 11.5 |  | 3.6 | 3.3 | 1.1 | 6.6 |  | 3.6 | 3.2 | 0.9 | 6.5 |
| SRP | µg /L |  | 65.8 | 44.1 | 24.9 | 207.6 |  | 21.7 | 18.2 | 4.8 | 49.9 |  | 21.8 | 18.0 | 5.1 | 53.4 |
| TP | µg /L |  | 104.9 | 84.5 | 35.5 | 214.5 |  | 44.1 | 41.6 | 10.9 | 82.6 |  | 43.3 | 40.3 | 11.4 | 87.3 |
| Cl^-^ | mg/L |  | 28.2 | 24.0 | 9.0 | 69.7 |  | 17.6 | 13.9 | 7.1 | 61.2 |  | 19.5 | 14.6 | 7.0 | 86.7 |
| SO_4_^2-^-S | mg/L |  | 31.3 | 17.6 | 5.5 | 174.3 |  | 27.9 | 11.1 | 4.8 | 172.3 |  | 26.8 | 10.9 | 4.8 | 176.5 |
| TOC | mg/L |  | 3.7 | 4.1 | 2.4 | 4.8 |  | 3.2 | 3.0 | 2.0 | 5.1 |  | 3.1 | 3.1 | 1.8 | 4.8 |
| DOC | mg/L |  | 3.1 | 3.2 | 2.0 | 4.4 |  | 2.7 | 2.5 | 1.7 | 4.4 |  | 2.7 | 2.5 | 1.5 | 4.4 |
| SiO_4_^4-^-Si | mg/L |  | 10.8 | 10.2 | 6.1 | 14.8 |  | 10.5 | 10.2 | 6.0 | 14.7 |  | 10.5 | 10.1 | 5.9 | 14.6 |
| TSS_river_ | mg/L |  | 6.9 | 6.0 | 2.0 | 16.6 |  | 6.5 | 6.5 | 2.0 | 10.7 |  | 6.5 | 6.2 | 2.4 | 11.2 |

**Table C2** Summary statistics of water chemistry variables measured from effluent at twelve wastewater treatment plants sampled in June 2013 and February 2014. TN, total nitrogen; SRP, soluble reactive phosphorus; TP, total phosphorus; TOC, total organic carbon; DOC, dissolved organic carbon; TSS, total suspended solids; SiO_4_^4-^-Si, Silica; TSS_river_, total suspended solids.

|  |  |  | Sampling date | | | | | | | | |
| --- | --- | --- | --- | --- | --- | --- | --- | --- | --- | --- | --- |
|  |  |  | June 2013 | | | |  | February 2014 | | | |
| Variable | Unit |  | Mean | Median | Min. | Max. |  | Mean | Median | Min. | Max. |
|  |  |  |  |  |  |  |  |  |  |  |  |
| Conductivity | µS/cm 20^o^ C |  | 846 | 859 | 560 | 1000 |  | 929 | 900 | 577 | 1355 |
| pH |  |  | 7.91 | 7.90 | 7.60 | 8.14 |  | 7.80 | 7.77 | 7.48 | 8.08 |
| Alkalinity | mmol/L |  | 4.32 | 4.34 | 2.95 | 5.62 |  | 4.61 | 4.39 | 3.25 | 6.48 |
| Hardness | mmol/L |  | 3.03 | 3.15 | 2.00 | 4.23 |  | 2.98 | 2.95 | 2.09 | 3.95 |
| Na^+^ | mg/L |  | 83.7 | 69.2 | 39.5 | 186.2 |  | 93.4 | 88.9 | 38.8 | 186.2 |
| K^+^ | mg/L |  | 13.2 | 13.1 | 8.5 | 20.1 |  | 12.6 | 12.6 | 6.1 | 17.8 |
| Ca^2+^ | mg/L |  | 98.9 | 104.5 | 57.6 | 134.2 |  | 96.1 | 91.4 | 66.8 | 123.0 |
| Mg^2+^ | mg/L |  | 12.9 | 12.9 | 8.0 | 20.3 |  | 12.5 | 11.8 | 8.1 | 19.8 |
| NH_4_^+^ | µg/L |  | 473 | 54 | 5 | 4930 |  | 1919 | 972 | 11 | 7025 |
| NO_2_^-^ | µg/L |  | 153 | 71 | 2 | 985 |  | 375 | 273 | 11 | 965 |
| NO_3_^-^ | mg/L |  | 17.2 | 12.2 | 6.4 | 32.7 |  | 14.4 | 14.0 | 3.8 | 30.3 |
| TN | mg/L |  | 17.6 | 12.5 | 6.9 | 36.3 |  | 18.8 | 20.2 | 4.3 | 35.9 |
| SRP | µg /L |  | 330 | 200 | 16 | 1193 |  | 551 | 129 | 10 | 4380 |
| TP | µg /L |  | 653 | 500 | 181 | 1625 |  | 684 | 261 | 46 | 4580 |
| Cl^-^ | mg/L |  | 116 | 102 | 48 | 211 |  | 118 | 98 | 60 | 203 |
| SO_4_^2-^-S | mg/L |  | 55 | 42 | 20 | 190 |  | 51 | 46 | 14 | 173 |
| TOC | mg/L |  | 8.3 | 6.7 | 4.2 | 16.3 |  | 6.0 | 5.9 | 3.7 | 9.1 |
| DOC | mg/L |  | 6.8 | 5.9 | 3.5 | 16.3 |  | 5.3 | 5.0 | 3.7 | 8.4 |
| SiO_4_^4-^-Si | mg/L |  | 14.9 | 15.3 | 10.3 | 21.9 |  | 14.5 | 14.4 | 9.8 | 18.9 |
| TSS_river_ | mg/L |  | 11.6 | 6.4 | 0.5 | 43.5 |  | 8.3 | 5.8 | 0.5 | 40.8 |

**Table C3** Results from principal components analysis (PCA) of mean wastewater composition from the twelve WWTPs sampled in June 2013 and March 2014. TN, total nitrogen; SRP, soluble reactive phosphorus; TP, total phosphorus; TOC, total organic carbon; DOC, dissolved organic carbon; SiO_4_^4-^-Si, Silica; TSS_river_, total suspended solids.

|  | Parameter | PC1 | PC2 | PC3 |
| --- | --- | --- | --- | --- |
|  |  |  |  |  |
| Variance explained (%) |  | 35.5 | 18.6 | 12.5 |
|  |  |  |  |  |
| Wastewater | Conductivity | -0.79 | 0.20 | 0.12 |
|  | pH | -0.53 | -0.32 | -0.08 |
|  | Alkalinity | -0.34 | 0.24 | -0.16 |
|  | Hardness | -0.66 | 0.42 | 0.22 |
|  | Na^+^ | -0.74 | 0.08 | -0.01 |
|  | K^+^ | -0.55 | -0.37 | -0.16 |
|  | Ca^2+^ | -0.66 | 0.32 | 0.17 |
|  | Mg^2+^ | -0.13 | 0.46 | 0.15 |
|  | NH_4_^+^ | -0.19 | -0.61 | 0.44 |
|  | NO_2_^-^ | -0.34 | -0.43 | 0.61 |
|  | NO_3_^-^ | -0.71 | -0.33 | 0.05 |
|  | TN | -0.72 | -0.28 | 0.17 |
|  | SRP | 0.08 | 0.63 | 0.32 |
|  | TP | -0.23 | 0.52 | 0.25 |
|  | Cl^-^ | -0.67 | -0.27 | -0.15 |
|  | SO_4_^2-^-S | -0.53 | 0.58 | 0.15 |
|  | TOC | -0.28 | 0.15 | -0.59 |
|  | DOC | -0.39 | 0.22 | -0.61 |
|  | SiO_4_^4-^-Si | -0.45 | -0.16 | -0.32 |
|  | TSS_river_ | -0.34 | -0.04 | -0.22 |

**C.3 Influence of habitat quality**

Sites were selected such as to minimize confounding effects due to differences of habitat conditions between the three locations at each site. Despite this procedure, it was impossible to avoid habitat differences completely (see Table C4). Therefore, we had to account for their effects on the composition of the macroinvertebrate communities. In short, we had to reduce the number of explanatory variables for these analyses, so the habitat characteristics were also reduced to three dimensions with a principal components analysis (PCA). We used these components to calculate the change in habitat between sampling locations (see Appendix B.3.3.2). The habitat PCA results are shown in Table C5.

Although the downstream change in taxa richness was significantly correlated with the change in habitat downstream (’ppcor’, *r* = -0.42; ‘hier.part’, *Z* = 1.75, P < 0.05; Table C9, Appendix C), this influence disappeared using rarefied taxa richness as the change response. Similarly, there was a weak correlation of wastewater dilution with the change in community evenness (’ppcor’, *r* = -0.42; ‘hier.part’, *Z* = 1.75, P < 0.05; Table C9, Appendix C), which may have been associated with the dominant proportions of oligochaetes at the downstream sampling locations.

**Table C4** Summary statistics of instream habitat variables measured at the sampling locations in the twelve streams. CPOM, coarse particulate organic matter; SMAC, submerged macrophytes; EMAC, emergent macrophytes; TSS, total suspendible sediment; ISS, suspendible inorganic sediment; SOS, suspendible organic sediment; %OS, % organic sediment.

|  |  |  |  | Sampling point | | | | | | | | | | | | | |
| --- | --- | --- | --- | --- | --- | --- | --- | --- | --- | --- | --- | --- | --- | --- | --- | --- | --- |
|  |  |  |  | D | | | |  | U1 | | | |  | U2 | | | |
| Category | Parameter | Unit |  | Mean | Med. | Min. | Max. |  | Mean | Med. | Min. | Max. |  | Mean | Med. | Min. | Max. |
|  |  |  |  |  |  |  |  |  |  |  |  |  |  |  |  |  |  |
| Inorganic | Bedrock | % |  | 13.3 | 11.7 | 0 | 30 |  | 18.3 | 20 | 0 | 40 |  | 15.0 | 12.5 | 0 | 30 |
|  | Boulder | % |  | 13.3 | 11.7 | 0 | 30 |  | 15.0 | 10 | 0 | 30 |  | 14.2 | 10 | 0 | 30 |
|  | Cobble | % |  | 32.5 | 31.3 | 10 | 40 |  | 33.3 | 31.7 | 10 | 40 |  | 34.2 | 37.1 | 10 | 40 |
|  | Gravel | % |  | 22.5 | 21.3 | 0 | 40 |  | 23.3 | 20 | 10 | 40 |  | 27.5 | 30 | 10 | 40 |
|  | Sand | % |  | 15.8 | 12.9 | 0 | 40 |  | 10.0 | 10 | 0 | 20 |  | 11.7 | 10 | 0 | 40 |
|  | Mud | % |  | 4.2 | 0 | 0 | 20 |  | 3.3 | 0 | 0 | 10 |  | 3.3 | 0 | 0 | 20 |
|  |  |  |  |  |  |  |  |  |  |  |  |  |  |  |  |  |  |
| Organic | CPOM | % |  | 8.3 | 10 | 0 | 20 |  | 7.5 | 10 | 0 | 20 |  | 9.2 | 10 | 0 | 20 |
|  | Algae | % |  | 3.3 | 0 | 0 | 40 |  | 4.2 | 0 | 0 | 30 |  | 5 | 0 | 0 | 30 |
|  | Bryophyte | % |  | 6.7 | 0 | 0 | 20 |  | 11.7 | 10 | 0 | 40 |  | 10 | 5 | 0 | 40 |
|  | SMAC | % |  | 0 | 0 | 0 | 0 |  | 7.5 | 3.8 | 0 | 30 |  | 11.7 | 10.8 | 0 | 40 |
|  | EMAC | % |  | 3.3 | 0 | 0 | 20 |  | 4.2 | 0 | 0 | 20 |  | 4.2 | 0 | 0 | 20 |
|  |  |  |  |  |  |  |  |  |  |  |  |  |  |  |  |  |  |
| Sediment | TSS | kg/m^2^ |  | 2.60 | 1.68 | 0.49 | 12.30 |  | 2.31 | 2.21 | 0.49 | 4.87 |  | 2.38 | 2.24 | 0.76 | 6.33 |
|  | ISS | kg/m^2^ |  | 2.33 | 1.47 | 0.42 | 11.13 |  | 2.08 | 2.01 | 0.42 | 4.27 |  | 2.13 | 2.00 | 0.65 | 5.58 |
|  | OSS | kg/m^2^ |  | 0.27 | 0.19 | 0.07 | 1.18 |  | 0.24 | 0.20 | 0.07 | 0.60 |  | 0.25 | 0.23 | 0.09 | 0.76 |
|  | %OS | % |  | 12.4 | 12.1 | 8.9 | 19.7 |  | 10.5 | 10.4 | 6.8 | 17.3 |  | 10.8 | 10.7 | 8.7 | 14.6 |
|  |  |  |  |  |  |  |  |  |  |  |  |  |  |  |  |  |  |
| Physical | Depth | m |  | 0.27 | 0.27 | 0.06 | 0.51 |  | 0.26 | 0.24 | 0.07 | 0.64 |  | 0.26 | 0.25 | 0.14 | 0.53 |
|  | Flow | m/s |  | 0.32 | 0.34 | 0.04 | 0.63 |  | 0.24 | 0.23 | 0.02 | 0.41 |  | 0.29 | 0.29 | 0.01 | 0.62 |
|  | Width | m |  | 3.9 | 4.0 | 2.0 | 5.8 |  | 3.7 | 3.8 | 1.6 | 6.8 |  | 3.3 | 3.3 | 2.0 | 4.6 |

**Table C5** Results from principal components analysis (PCA) of instream habitat variables at sampling locations (D, U1, U2). The site scores from this analysis were used to generate the estimate of habitat change at the downstream sites. CPOM, coarse particulate organic matter; EMAC, emergent macrophytes; TSS, log-transformed total benthic suspendible sediment.

|  | Parameter | PC1 | PC2 | PC3 |
| --- | --- | --- | --- | --- |
|  |  |  |  |  |
| Variance explained (%) |  | 19.0 | 15.8 | 13.4 |
|  |  |  |  |  |
| Instream habitat | Bedrock | -0.02 | 0.58 | 0.63 |
|  | Boulder | -0.62 | -0.49 | -0.25 |
|  | Cobble | 1.04 | -0.14 | -0.14 |
|  | Gravel | 0.80 | 0.20 | -0.15 |
|  | Sand | -0.06 | 0.53 | -0.72 |
|  | Mud | 0.83 | 0.21 | 0.20 |
|  | CPOM | -0.61 | 0.74 | 0.07 |
|  | Algae | 0.38 | 0.10 | 0.24 |
|  | Bryophyte | -0.45 | -0.57 | -0.21 |
|  | EMAC | 0.29 | -0.64 | -0.71 |
|  | TSS | 0.27 | -0.61 | 0.51 |
|  | Depth | 0.17 | -0.30 | 0.89 |
|  | Flow | -0.59 | -0.61 | 0.45 |
|  | Width | 0.37 | -0.66 | -0.11 |

**C.4 Community composition**

**C.4.1 Taxa composition**

Overall, a total of 74 taxa from 18 different orders (classes) were recorded from the 12 study streams (Table C7). The most taxa rich order was the Diptera (14), followed by the Trichoptera (13). In the pollution-sensitive EPT (Ephemeroptera, Plecoptera, Trichoptera) group of taxa, a total of 27 taxa were recorded, with 7 Ephemeroptera and 7 Plecoptera families, in addition to the 13 Trichopteran families stated above.

Invertebrate communities at upstream sampling locations were generally dominated by chironomid dipterans, baetids mayflies, and gammarid amphipods (Table C7). Other abundant taxa included oligochaete worms, elmid beetles, and simullid dipterans. Of the pollution-sensitive EPT (Ephemeroptera, Plectoptera, Trichoptera) fauna, baetid mayflies were the most abundant and commonly recorded. Other common EPT taxa included leptophlebiid and heptageniid mayflies, leuctrid and nemourid stoneflies, and limnephilid, hyrdopsychid and rhyacophilid caddisflies, although none of these taxa were highly abundant (Table C7).

**C.4.2 Effects of land use and water quality on community composition**

*C.4.2.1 Upstream influences of landuse on community composition*

The partial redundancy analyses (pRDA) of upstream communities show clear relationships with land use in the catchments (see Fig. C1).

*C.4.2.2 Outlying site in unconstrained ordination analysis (NMDS)*

Across all streams and sampling locations, one study site (Kernenried) appeared as a clear outlier in a NMDS plot (Fig. C2). This was due to exceptionally high proportions of gammarid amphipods at all three sampling locations.

*C.4.2.3 Effects of wastewater on additional community descriptors*

Inputs of wastewater appeaed to greatly increase raw abundances of invertebrates, although this influence dissipated when the oligochaetes were removed (Table C6). There was no significant effect of wastewater on the richness of EPT families, although relative abundances were greatly reduced (Table C6). This again likely reflected the dominance of the oligochate worms at wastewater-impacted sites, reinforced by the significant increase in the Berger-Parker dominance index (Table C6) at downstreams sites (D). The Swiss IBCH was insensitive to wastewater impacts, suggesting this indicator is not useful for detecting pollution-induced changes in streams with background (upstream) levels of anthropogenic pressure (Table C6).

*C.4.2.4 Excluding oligochaetes from NMDS analyses*

The only significant effects of wastewater inputs observed when excluding the oligochaete worms was a significant difference between sampling location D and U2 (NMDS, Fig. C3A, ‘adonis’, *F_2,32_* = 0.375, *R^2^* = 0.02, *P* > 0.05). The lack of a systematic response may have been exacerbated by the wide range of streams sampled, and although there was a moderately-sized effect on the community change downstream (minus Oligochates), this change was not significant (Table C8). This contrasted with the large effect size and highly significant result when considering the total community (Table C8).

*C.4.2.5 Taxonomic-based community descriptors and multiple-regression models*

The results of the multiple-regression models describe how all influencing factors (including wastewater) are correlated with the observed changes in macroinvertebrate communities downstream of the WWTP. There were few consistent (and statistically significant) relationships between predictor variables and taxonomic-based metrics of community composition (Table C9).

*C.4.2.6 Trait-based community descriptors and multiple-regression models*

However, more subtle effects could be detected by using the two trait-based indices, where the SPEAR index was able to discern changes that differed from the total community and Saprobic Index. In this context, we removed one site, Hornussen, from the regression analyses. It was identified as an outlier after plotting the change in the SPEAR index with wastewater dilution factors (i.e., *Q_347_/Q_ww_*), calculated for the 95^th^ percentile of discharge in the streams (*Q_347_*_;_ Fig. 6D, Main text). We justified its removal by plotting the same response variable and mean stream discharges (*Q_avg_*; Fig. C5B), which showed the same pattern, albeit with Hornussen no longer an outlier. It appears that Hornussen may have a ‘flashy’ hydrograph, characterised by large, infrequent floods, meaning its *Q_avg_* is not congruent with *Q_347_* (Fig. C5A). Studies have shown that the effects of hydrodynamic disturbance can override the impacts of wastewater inputs in streams by “resetting” community composition between upstream and downstream sites ([Ortiz & Puig 2007](#_ENREF_18)). This may help explain the low level of change observed downstream of the wastewater discharge in Hornussen. Its removal did not materially affect the results of the regression, but it did help improve the fit.

*C.4.2.7 Additional outlying sites*

There was also a clear outlier when comparing the upstream saprobic index with total community change. One site (Niederdorf) showed by far the most extreme total community change between upstream and downstream despite a moderate saprobic condition upstream (Fig. C6). It has been reported that this WWTP suffers from periodic technical problems (i.e., industrial pollution entering with the raw effluent, thus altering the composition of the treated wastewater). It is probable that the toxicants associated with these stochastic events and therefore not accounted for in our analyses may have caused the strong difference between upstream and downstream communities at this site.

**C.4.3 Beta-diversity partitioning**

Beta-diversity partitioning analysis showed that there were no significant differences in community dissimilarity between the two upstream reference locations (U1, U2) and the contrast between U1 and the downstream, wastewater-impacted location (D) using taxa occupancy data (Fig. C4). Sørensen’s dissimilarity index did not significantly change for the different contrasts (U1,U2) and (D,U1) across 12 sites (*F_1,11_* = 1.75, *P* = 0.213). The mean turnover (taxa replacement) component (Simpson’s dissimilarity) did not differ significantly (*F_1,11_* = 1.42, *P* = 0.259); nor did the mean nestedness component (taxa loss) of Sørensen dissimilarity (*F_1,11_* = 1.61, *P* = 0.231). See Appendix B.3.4 for a full description of methods.

Table C6 Additional taxonomic-based indicators of stream condition at sampling points downstream (D) and upstream (U1,U2) of wastewater inputs using macroinvertebrate community data collected from twelve Swiss streams. Mean values are presented ± 1 standard deviation. Cohen’s *d* quantifies differences in invertebrate community metrics downstream (D) compared to upstream (U1,U2). F-statistics, degrees of freedom, P-values, and the proportion of variance explained by the random factor (Stream) are presented from mixed-model ANOVAs where the sampling point (U1,U2,D) was the fixed factor. For a full description of indicators, see Appendix B.2.3.

| Indicator | Sampling point | | |  |  |  |  | Stream  % var. |
| --- | --- | --- | --- | --- | --- | --- | --- | --- |
|  | U2 | U1 | D | Cohen’s *d* | *F-*stat | d.f. | Significance |  |
| Abundance | 2142 ± 943 | 2386 ± 1698 | 3944 ± 2777 | 0.77 | 6.93 | 2, 24 | *P* < 0.01 | 46 |
| Abund. minus Oligochaetes | 1964 ± 897 | 2150 ± 1663 | 2256 ± 2569 | 0.10 | 0.25 | 2, 24 | *P* = 0.779 | 58 |
| EPT_family_ richness | 9.3 ± 3.2 | 9.9 ± 3.4 | 9.6 ± 3.8 | -0.01 | 1.24 | 2, 24 | *P =* 0.308 | 92 |
| % EPT | 27.3 ± 15.8 | 24.0 ± 16.7 | 11.7 ± 7.9 | -1.10 | 11.9 | 2, 24 | *P* < 0.001 | 50 |
| % EPT minus Oligochaetes | 30.1 ± 17.5 | 27.3 ± 18.0 | 23.8 ± 14.6 | -0.30 | 2.54 | 2, 24 | *P =* 0.100 | 80 |
| Berger-Parker | 42.0 ± 13.2 | 42.6 ± 14.0 | 56.1 ± 15.1 | 0.97 | 12.8 | 2, 24 | *P* < 0.001 | 54 |
| IBCH | 13.4 ± 3.1 | 13.7 ± 2.3 | 13.2 ± 3.4 | -0.12 | 0.49 | 2, 24 | *P =* 0.617 | 81 |

Table C7 Taxa relative abundances at sampling points downstream (D) and upstream (U1,U2) of wastewater discharges using macroinvertebrate community data collected from twelve Swiss streams sampled during February and March 2013. The status of SPEAR fauna (i.e, 1, SPEAR taxa; 0, non-SPEAR taxa; NA = no designation available) is indicated, as too are the taxa-specific Saprobic Index scores used. Mean relative abundances are presented ± 1 standard deviation (SD), and counts indicate the number of sites a taxon was recorded for each sampling location (U2,U1,D). NA, value not available.

| Order | Taxa | SPEAR taxa | Saprobic score |  | Sampling location | | | | | | | | |
| --- | --- | --- | --- | --- | --- | --- | --- | --- | --- | --- | --- | --- | --- |
|  |  |  |  |  |  | U2 |  |  | U1 |  |  | D |  |
|  |  |  |  |  | Mean | SD | Count | Mean | SD | Count | Mean | SD | Count |
|  |  |  |  |  |  |  |  |  |  |  |  |  |  |
| Ephemeroptera | Baetidae | 1 | 1.86 |  | 18.81 | 12.87 | 12 | 15.86 | 12.36 | 12 | 7.70 | 6.37 | 12 |
|  | Caenidae | 1 | 2.00 |  | 0.02 | 0.05 | 2 | 0.01 | 0.04 | 1 | 0.00 | 0.00 | 0 |
|  | Ephemerellidae | 1 | 1.80 |  | 1.49 | 3.57 | 6 | 1.21 | 3.53 | 5 | 1.02 | 2.16 | 7 |
|  | Ephemeridae | 1 | 2.03 |  | 0.05 | 0.12 | 2 | 0.01 | 0.05 | 1 | 0.01 | 0.02 | 1 |
|  | Heptageniidae | 1 | 1.52 |  | 1.20 | 1.81 | 7 | 0.98 | 1.60 | 9 | 0.32 | 0.39 | 8 |
|  | Leptophlebiidae | 1 | 1.69 |  | 1.56 | 2.21 | 9 | 1.44 | 3.10 | 9 | 0.54 | 1.06 | 8 |
|  | Siphlonuridae | 1 | 2.00 |  | 0.00 | 0.00 | 0 | 0.00 | 0.00 | 0 | 0.01 | 0.04 | 1 |
| Plecoptera | Capniidae | 1 | 1.30 |  | 0.00 | 0.00 | 0 | 0.00 | 0.00 | 0 | 0.01 | 0.03 | 1 |
|  | Chloroperlidae | 1 | 1.23 |  | 0.01 | 0.05 | 1 | 0.02 | 0.05 | 2 | 0.00 | 0.00 | 0 |
|  | Leuctridae | 1 | 1.19 |  | 1.49 | 2.69 | 8 | 1.51 | 2.96 | 7 | 0.65 | 1.47 | 8 |
|  | Nemouridae | 1 | 1.22 |  | 0.76 | 0.96 | 9 | 0.58 | 0.88 | 8 | 0.29 | 0.46 | 8 |
|  | Perlidae | 1 | 1.30 |  | 0.00 | 0.00 | 0 | 0.00 | 0.00 | 0 | 0.00 | 0.01 | 1 |
|  | Perlodidae | 1 | 1.20 |  | 0.02 | 0.04 | 3 | 0.03 | 0.05 | 4 | 0.02 | 0.07 | 3 |
|  | Taeniopterygidae | 1 | 1.27 |  | 0.07 | 0.14 | 4 | 0.04 | 0.07 | 4 | 0.04 | 0.10 | 5 |
| Trichoptera | Glossosomatidae | 1 | 1.14 |  | 0.00 | 0.00 | 0 | 0.00 | 0.00 | 0 | 0.01 | 0.02 | 1 |
|  | Goeridae | 1 | 1.45 |  | 0.05 | 0.14 | 2 | 0.08 | 0.27 | 2 | 0.00 | 0.00 | 1 |
|  | Helicopsychidae | 1 | 1.14 |  | 0.03 | 0.09 | 1 | 0.00 | 0.00 | 0 | 0.00 | 0.00 | 0 |
|  | Hydropsychidae | 0 | 1.78 |  | 0.71 | 0.94 | 9 | 0.85 | 1.17 | 11 | 0.59 | 0.82 | 11 |
|  | Hydroptilidae | 1 | 2.00 |  | 0.02 | 0.06 | 2 | 0.04 | 0.06 | 5 | 0.03 | 0.07 | 4 |
|  | Lepidostomatidae | 1 | 1.53 |  | 0.00 | 0.00 | 0 | 0.00 | 0.00 | 0 | 0.00 | 0.00 | 1 |
|  | Leptoceridae | 1 | 2.06 |  | 0.03 | 0.11 | 1 | 0.06 | 0.20 | 1 | 0.02 | 0.06 | 1 |
|  | Limnephilidae | 1 | 1.47 |  | 0.45 | 0.52 | 12 | 0.76 | 1.34 | 11 | 0.28 | 0.31 | 12 |
|  | Odontoceridae | 1 | 1.40 |  | 0.03 | 0.07 | 3 | 0.03 | 0.04 | 5 | 0.02 | 0.04 | 3 |
|  | Polycentropodidae | 1 | 1.63 |  | 0.04 | 0.12 | 2 | 0.04 | 0.08 | 4 | 0.02 | 0.03 | 5 |
|  | Psychomyiidae | 1 | 1.72 |  | 0.07 | 0.14 | 5 | 0.08 | 0.17 | 5 | 0.04 | 0.06 | 4 |
|  | Rhyacophilidae | 1 | 1.27 |  | 0.37 | 0.49 | 10 | 0.38 | 0.48 | 11 | 0.08 | 0.11 | 7 |
|  | Sericostomatidae | 1 | 1.63 |  | 0.02 | 0.04 | 2 | 0.02 | 0.04 | 2 | 0.01 | 0.02 | 2 |
| Megaloptera | Sialidae | 1 | 2.25 |  | 0.02 | 0.04 | 3 | 0.00 | 0.00 | 0 | 0.00 | 0.00 | 0 |
| Odonata | Calopterygidae | 1 | 2.00 |  | 0.01 | 0.04 | 1 | 0.02 | 0.04 | 2 | 0.01 | 0.02 | 2 |
|  | Coenagrionidae | 1 | 1.75 |  | 0.02 | 0.06 | 1 | 0.00 | 0.00 | 0 | 0.00 | 0.00 | 0 |
|  | Cordulegasteridae | 0 | 1.50 |  | 0.00 | 0.00 | 0 | 0.00 | 0.00 | 0 | 0.00 | 0.02 | 1 |
|  | Gomphidae | 0 | 2.00 |  | 0.01 | 0.02 | 1 | 0.00 | 0.00 | 0 | 0.00 | 0.00 | 0 |
| Coleoptera | Dryopidae | 0 | 1.90 |  | 0.00 | 0.01 | 1 | 0.01 | 0.03 | 1 | 0.00 | 0.00 | 0 |
|  | Dytiscidae | 0 | 2.60 |  | 0.01 | 0.03 | 3 | 0.01 | 0.04 | 1 | 0.02 | 0.04 | 4 |
|  | Elmidae | 0 | 1.90 |  | 6.88 | 6.48 | 10 | 5.97 | 5.78 | 10 | 2.99 | 3.12 | 10 |
|  | Gyrinidae | 0 | 1.90 |  | 0.02 | 0.03 | 3 | 0.02 | 0.05 | 3 | 0.01 | 0.02 | 1 |
|  | Haliplidae | 0 | 1.90 |  | 0.01 | 0.03 | 2 | 0.03 | 0.09 | 1 | 0.01 | 0.04 | 1 |
|  | Hydraenidae | 0 | 1.50 |  | 0.05 | 0.06 | 6 | 0.04 | 0.07 | 4 | 0.01 | 0.02 | 3 |
|  | Scirtidae | 0 | 1.60 |  | 0.02 | 0.04 | 2 | 0.04 | 0.10 | 3 | 0.00 | 0.00 | 0 |
| Diptera | Athericidae | 1 | 1.50 |  | 0.00 | 0.00 | 0 | 0.02 | 0.07 | 1 | 0.00 | 0.01 | 1 |
|  | Ceratopogonidae | 0 | 1.76 |  | 0.90 | 0.97 | 11 | 1.34 | 1.88 | 10 | 0.69 | 0.62 | 11 |
|  | Chironomidae | 0 | 1.76 |  | 27.87 | 17.12 | 12 | 30.19 | 17.26 | 12 | 20.10 | 12.10 | 12 |
|  | Dixidae | 1 | 1.50 |  | 0.00 | 0.00 | 0 | 0.00 | 0.01 | 1 | 0.00 | 0.00 | 0 |
|  | Empididae | 1 | 0.40 |  | 0.68 | 1.47 | 9 | 0.79 | 1.09 | 10 | 0.31 | 0.43 | 10 |
|  | Limoniidae | 1 | 1.50 |  | 0.46 | 0.52 | 10 | 0.58 | 0.52 | 12 | 0.31 | 0.47 | 10 |
|  | Muscidae | 1 | 2.00 |  | 0.00 | 0.00 | 0 | 0.01 | 0.02 | 1 | 0.01 | 0.01 | 2 |
|  | Psychodidae | 0 | 1.00 |  | 0.04 | 0.06 | 6 | 0.11 | 0.13 | 9 | 0.12 | 0.31 | 6 |
|  | Ptychopteridae | 1 | 1.50 |  | 0.06 | 0.19 | 1 | 0.00 | 0.00 | 0 | 0.00 | 0.00 | 0 |
|  | Simuliidae | 0 | 1.57 |  | 5.41 | 8.08 | 12 | 5.34 | 6.29 | 12 | 2.88 | 2.82 | 12 |
|  | Stratiomyidae | NA | 3.00 |  | 0.03 | 0.11 | 1 | 0.08 | 0.23 | 3 | 0.00 | 0.00 | 1 |
|  | Syrphidae | 0 | 4.00 |  | 0.00 | 0.00 | 0 | 0.01 | 0.03 | 1 | 0.00 | 0.00 | 0 |
|  | Tabanidae | 0 | 1.40 |  | 0.00 | 0.00 | 0 | 0.00 | 0.00 | 0 | 0.00 | 0.00 | 1 |
|  | Tipulidae | 0 | 1.50 |  | 0.06 | 0.10 | 6 | 0.02 | 0.06 | 2 | 0.05 | 0.14 | 3 |
| Heteroptera | Aphelocheiridae | 0 | 2.00 |  | 0.00 | 0.01 | 1 | 0.00 | 0.00 | 0 | 0.00 | 0.00 | 0 |
|  | Corixidae | 0 | 1.50 |  | 0.02 | 0.06 | 3 | 0.06 | 0.22 | 1 | 0.00 | 0.00 | 0 |
|  | Veliidae | 0 | 1.50 |  | 0.00 | 0.00 | 0 | 0.00 | 0.01 | 1 | 0.00 | 0.00 | 0 |
| Amphipoda | Gammaridae | 0 | 1.98 |  | 17.14 | 22.02 | 12 | 14.78 | 21.58 | 12 | 10.39 | 23.61 | 12 |
| Arachnida | Hydracarina | 0 | 1.50 |  | 1.73 | 1.46 | 10 | 1.70 | 1.45 | 10 | 0.86 | 0.91 | 10 |
| Isopoda | Asellidae | 0 | 2.80 |  | 0.14 | 0.22 | 5 | 0.20 | 0.46 | 4 | 0.13 | 0.22 | 6 |
| Bivalvia | Corbiculidae | 0 | 2.20 |  | 0.00 | 0.00 | 0 | 0.00 | 0.00 | 0 | 0.07 | 0.24 | 1 |
|  | Sphaeriidae | 0 | 2.13 |  | 1.06 | 2.31 | 9 | 0.91 | 1.87 | 8 | 0.87 | 2.38 | 9 |
| Gastropoda | Ancylidae | 0 | 1.90 |  | 0.01 | 0.02 | 1 | 0.00 | 0.01 | 1 | 0.01 | 0.02 | 1 |
|  | Hydrobiidae | 0 | 2.00 |  | 0.03 | 0.06 | 3 | 0.02 | 0.03 | 3 | 0.03 | 0.07 | 4 |
|  | Lymnaeidae | 0 | 2.30 |  | 0.04 | 0.08 | 3 | 0.05 | 0.09 | 4 | 0.06 | 0.09 | 4 |
|  | Physidae | 0 | 2.40 |  | 0.01 | 0.02 | 2 | 0.01 | 0.03 | 1 | 0.01 | 0.02 | 1 |
|  | Planorbidae | 0 | 2.08 |  | 0.04 | 0.15 | 1 | 0.08 | 0.27 | 1 | 0.02 | 0.06 | 1 |
|  | Valvatidae | 0 | 2.00 |  | 0.00 | 0.01 | 1 | 0.00 | 0.00 | 0 | 0.00 | 0.00 | 1 |
| Cnidaria | Cnidaria | NA | 1.70 |  | 0.12 | 0.42 | 1 | 0.13 | 0.44 | 1 | 0.04 | 0.14 | 2 |
| Hirudinea | Erpobdellidae | 0 | 2.43 |  | 0.06 | 0.10 | 5 | 0.03 | 0.08 | 2 | 0.05 | 0.09 | 4 |
|  | Glossiphoniidae | 0 | 2.32 |  | 0.01 | 0.04 | 2 | 0.04 | 0.09 | 2 | 0.02 | 0.05 | 3 |
| Nematoda | Nematoda | 0 | 3.02 |  | 0.50 | 1.36 | 8 | 0.35 | 0.78 | 9 | 0.74 | 2.11 | 6 |
| Oligochaeta | Oligochaeta | 0 | 3.02 |  | 8.67 | 3.88 | 12 | 12.08 | 9.61 | 12 | 46.95 | 20.96 | 12 |
| Platyhelminthes | Dugesiidae | 0 | 2.00 |  | 0.22 | 0.62 | 5 | 0.34 | 0.81 | 4 | 0.50 | 1.04 | 6 |
|  | Planariidae | 0 | 1.40 |  | 0.35 | 0.76 | 4 | 0.54 | 1.24 | 3 | 0.02 | 0.06 | 2 |
|  |  |  |  |  |  |  |  |  |  |  |  |  |  |

**
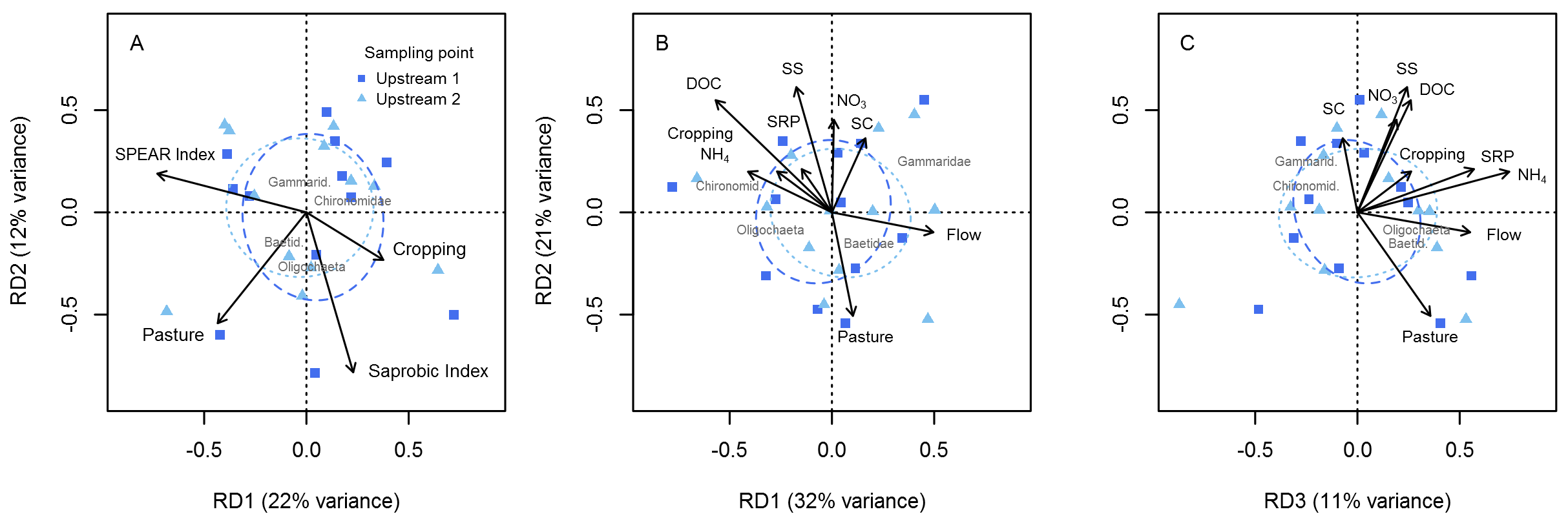
**

**Figure C1** Results from partial redundancy analyses (pRDA) of upstream invertebrate community data, catchment properties, and local environmental factors. A) show RD1 and RD2 site scores (weighted sums of species scores) from the first upstream pRDA model indicating the correlation of two trait-based invertebrate indices (Saprobic, SPEAR pesticides) and catchment landuses that significantly explain variation in community composition. The results from the second upstream pRDA model including local environmental variables (water quality, habitat) is shown in B) with RD1 and RD2, whereas C) shows RD3 and RD2. Standard dispersion ellipses represent 95% confidence intervals and the position of common invertebrate taxa is indicated in all plots. Arable cropping and pasture (% area in upstream catchment); Flow, average water velocities (m/s); NH_4_, ammonia (µg/L); SRP, soluble reactive phosphorus (µg/L); NO_3_, nitrate (mg/L); SC, specific conductivity (µS/cm 20^°^ C); DOC, dissolved organic carbon (mg/L); SS, suspended sediment (mg/L).


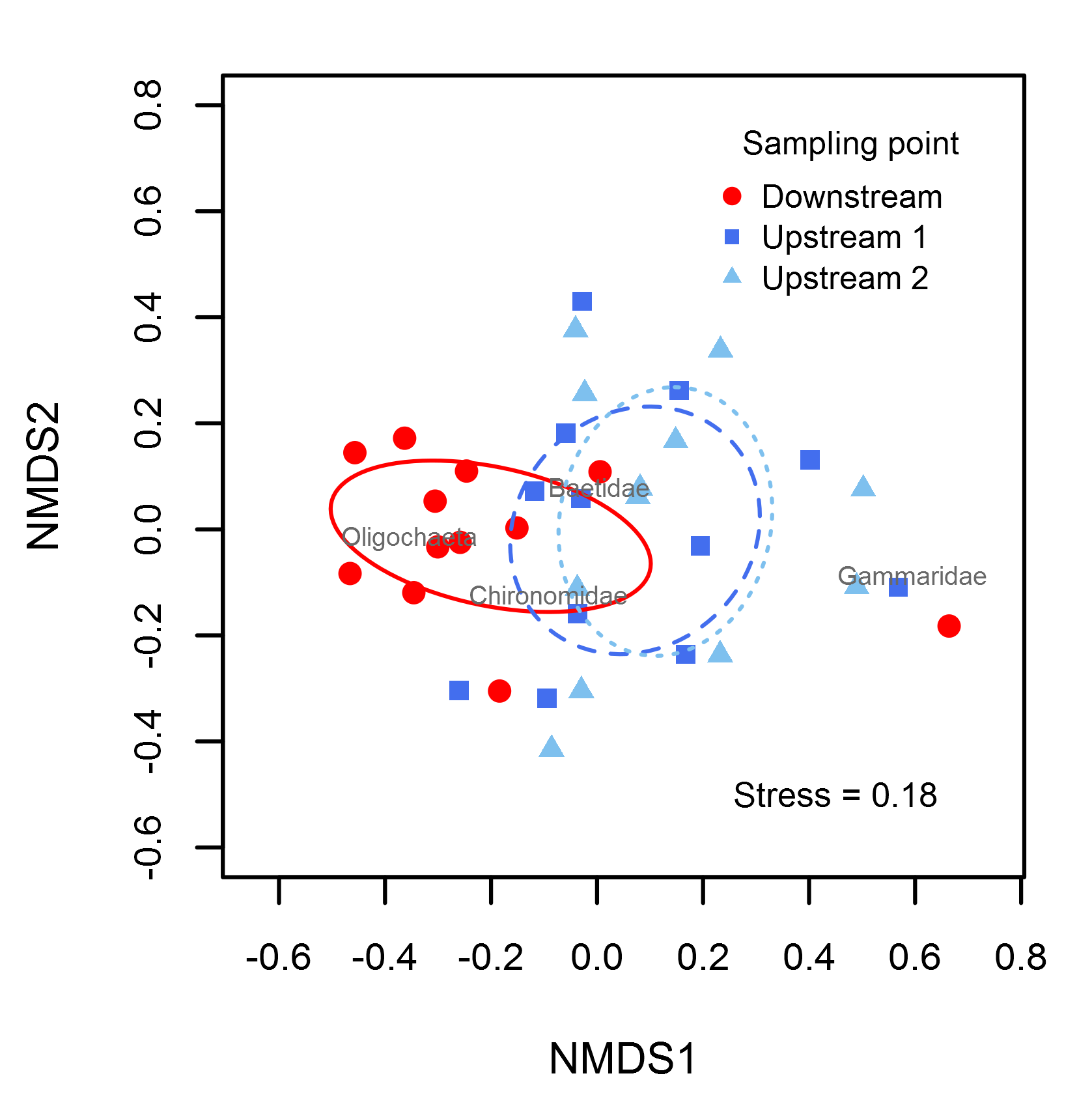


**Figure C2** Results from non-metric dimensional scaling (NMDS) analysis of invertebrate community relative abundance data from all 12 streams sampled. The presence of an outlying site (Kernenried) due to high relative abundances of gammarid amphipods is seen on the right-hand side of the plot. Standard dispersion ellipses represent 95% confidence intervals, and the position of common invertebrate taxa is indicated.


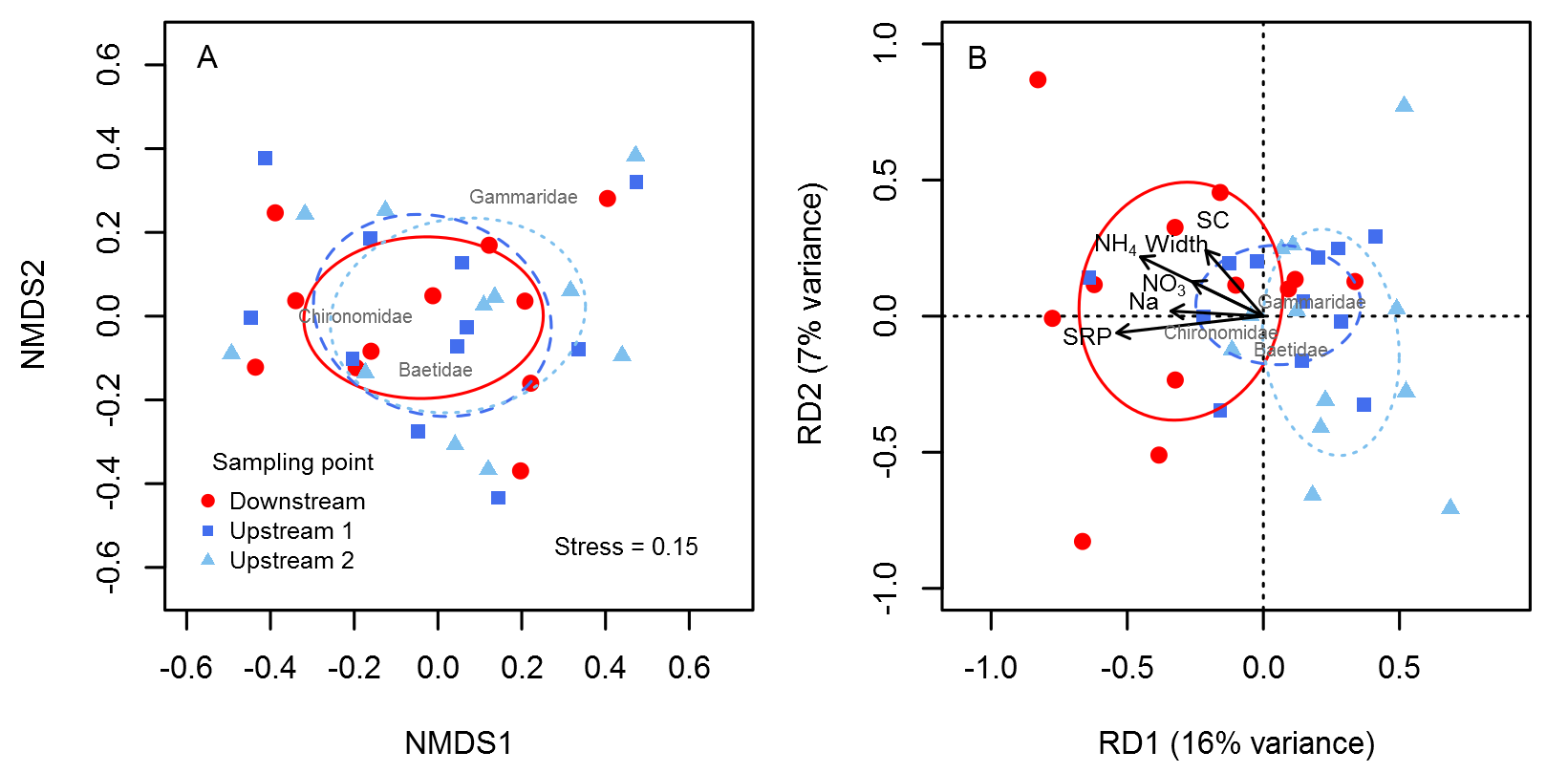


**Figure C3** Results from analyses of invertebrate community relative abundance data (minus Oligochaeta) using A) non-metric dimensional scaling (NMDS); B) partial redundancy analysis (pRDA) and physicochemical variables. Standard dispersion ellipses represent 95% confidence intervals, and the position of common invertebrate taxa is indicated in all plots. SC, specific conductivity (µS/cm 20^°^ C); NH_4_, ammonia (µg/L); SRP, soluble reactive phosphorus (µg/L); Na, Sodium (mg/L); NO_3_, nitrate (mg/L); Width, stream width.

**Table C8** Change in invertebrate taxonomic composition between sampling points downstream (D) and upstream (U1, U2) of wastewater inputs using macroinvertebrate community data (relative abundances) collected from twelve Swiss streams sampled during Spring 2013. Described here is the change indice using the NMDS site scores to calculate the Euclidean distance between sampling location at each site (i.e., distance in 2-dimensions). Mean values are presented ± 1 standard deviation. Cohen’s *d* quantifies the difference in invertebrate community change downstream (U2-D,U1-D) compared to the null expectation (i.e., upstream change U2-U2). F-statistics, degrees of freedom, P-values, and the proportion of variance explained by the random factor (stream) are presented from mixed-model ANOVAs where the sampling point contrast (U2-U1, U2-D, U1-D) was the fixed factor.

|  | Sampling location comparision | | |  |  |  |  | Stream |
| --- | --- | --- | --- | --- | --- | --- | --- | --- |
| Community NMDS | U2-U1 | U2-D | U1-D | Cohen’s *d* | *F-*stat | d.f. | Significance | % var. |
| Total | 0.120 ± 0.063 | 0.394 ± 0.213 | 0.341 ± 0.226 | 1.49 | 12.2 | 2, 24 | *P* < 0.001 | 22 |
| Minus Oligochaetes | 0.101 ± 0.069 | 0.146 ± 0.090 | 0.133 ± 0.070 | 0.49 | 1.56 | 2, 24 | *P* = 0.231 | 24 |

**Table C9** Results from multiple-regression models of invertebrate diversity change responses using hierchical partitioning. Shown are the independent (I), joint (J) and total effects of predictors on the response variables. I% represents the contribution of the I-values to the total explained variance in the response variables. The partial correlation coefficient (pcor) indicates the nature of the top predictor variables relationship against the response variable. For a full description of response and predictor variables, see the Table 1, Main text and Appendix B.2.

| **Response** | **Predictor** | **I** | **J** | **Total** | **%I** | **Obs** | ***Z*-score** | **pcor** |
| --- | --- | --- | --- | --- | --- | --- | --- | --- |
|  |  |  |  |  |  |  |  |  |
| ∆ Taxa | PC_habitat_ | 0.276 | 0.153 | 0.429 | 56.1 | 0.28 | 1.75* | -0.42^ns^ |
| (richness) | DF_ww_ | 0.101 | 0.082 | 0.183 | 20.6 | 0.10 | -0.03 |  |
|  | RD1_invert_ | 0.048 | 0.053 | 0.101 | 9.7 | 0.05 | -0.49 |  |
|  | RD2_invert_ | 0.025 | -0.002 | 0.023 | 5.2 | 0.03 | -0.76 |  |
|  | RD3_invert_ | 0.025 | -0.009 | 0.016 | 5.1 | 0.02 | -0.74 |  |
|  | PC1_ww_ | 0.016 | 0.014 | 0.030 | 3.3 | 0.02 | -0.84 |  |
|  |  |  |  |  |  |  |  |  |
| ∆ Rarefied | PC_habitat_ | 0.220 | -0.044 | 0.176 | 25.8 | 0.22 | 1.27 | 0.79† |
| (taxa richness) | RD1_invert_ | 0.175 | 0.052 | 0.227 | 20.5 | 0.18 | 0.79 |  |
|  | RD2_invert_ | 0.152 | -0.139 | 0.013 | 17.8 | 0.15 | 0.45 |  |
|  | DF_ww_ | 0.143 | 0.016 | 0.160 | 16.8 | 0.14 | 0.42 |  |
|  | PC1_ww_ | 0.095 | 0.035 | 0.130 | 11.1 | 0.09 | -0.07 |  |
|  | RD3_invert_ | 0.068 | -0.063 | 0.005 | 8.0 | 0.07 | -0.30 |  |
|  |  |  |  |  |  |  |  |  |
| ∆ Evenness | DF_ww_ | 0.285 | 0.048 | 0.333 | 50.4 | 0.28 | 1.81* | -0.59^ns^ |
|  | RD3_invert_ | 0.097 | 0.053 | 0.150 | 17.1 | 0.10 | -0.05 |  |
|  | RD1_invert_ | 0.094 | -0.014 | 0.079 | 16.5 | 0.09 | -0.07 |  |
|  | PC_habitat_ | 0.039 | -0.009 | 0.031 | 6.9 | 0.04 | -0.58 |  |
|  | RD2_invert_ | 0.037 | -0.020 | 0.017 | 6.6 | 0.04 | -0.60 |  |
|  | PC1_ww_ | 0.014 | 0.006 | 0.020 | 2.5 | 0.01 | -0.82 |  |
|  |  |  |  |  |  |  |  |  |
| ∆ Fisher’s | RD2_invert_ | 0.252 | 0.056 | 0.308 | 31.4 | 0.25 | 1.53 | 0.57^ns^ |
|  | PC_habitat_ | 0.208 | -0.033 | 0.176 | 25.9 | 0.21 | 1.11 |  |
|  | DF_ww_ | 0.131 | 0.033 | 0.164 | 16.3 | 0.13 | 0.27 |  |
|  | RD1_invert_ | 0.111 | -0.106 | 0.005 | 13.8 | 0.11 | 0.10 |  |
|  | RD3_invert_ | 0.054 | -0.051 | 0.003 | 6.8 | 0.05 | -0.46 |  |
|  | PC1_ww_ | 0.047 | 0.019 | 0.066 | 5.8 | 0.05 | -0.52 |  |
|  |  |  |  |  |  |  |  |  |
| ∆ Community | RD3_invert_ | 0.563 | 0.039 | 0.601 | 70.6 | 0.56 | 4.39* | -0.85† |
|  | RD1_invert_ | 0.127 | 0.026 | 0.153 | 15.9 | 0.13 | 0.25 |  |
|  | PC_habitat_ | 0.042 | -0.037 | 0.005 | 5.2 | 0.04 | -0.57 |  |
|  | RD2_invert_ | 0.037 | 0.001 | 0.038 | 4.6 | 0.04 | -0.61 |  |
|  | PC1_ww_ | 0.015 | -0.013 | 0.002 | 1.9 | 0.01 | -0.80 |  |
|  | DF_ww_ | 0.014 | 0.009 | 0.023 | 1.8 | 0.01 | -0.82 |  |
|  |  |  |  |  |  |  |  |  |

**
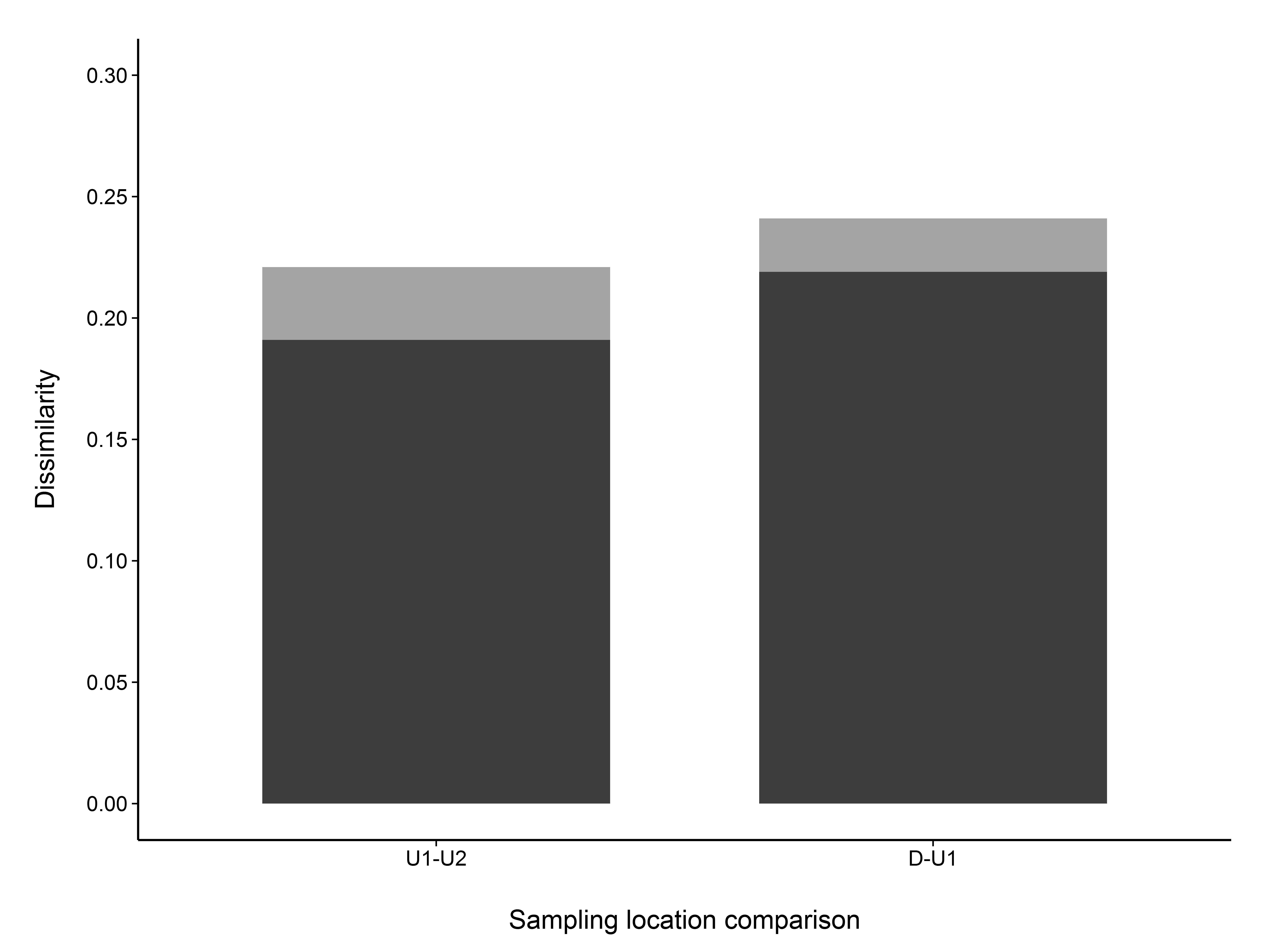
**

**Figure C4** Beta-diversity partitioning analysis showed that there were no significant differences in community dissimilarity between the two upstream reference locations (U1-U2) and the contrast between U1 and the downstream, wastewater-impacted location (D-U1) using taxa occupancy data (i.e., presence/absence). The mean dissimilarity values from 12 sites for the different contrasts (U1-U2) and (D-U1) represents Sørensen’s dissimilarity index (*F_1,11_* = 1.75, *P* = 0.213). The area shaded in dark grey indicates the mean turnover (taxa replacement) component (Simpson’s dissimilarity; *F_1,11_* = 1.42, *P* = 0.259), and the area in light grey shows the mean nestedness component (taxa loss) of Sørensen dissimilarity (*F_1,11_* = 1.61, *P* = 0.231).

See Appendix B.3.4 for a full description of methods used.


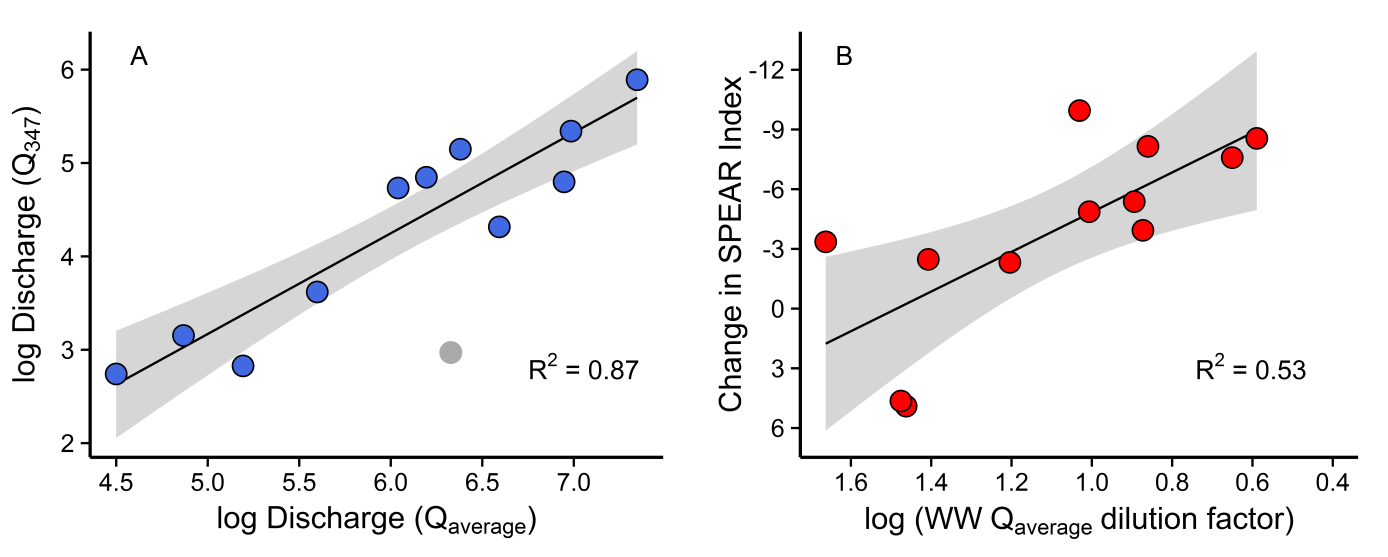


**Figure C5** A) The linear regression of the log-transformed mean stream discharge (*Q_average_*) and the 95^th^ percentile of stream discharge (*Q_347_*) over a ten-year period shows a strong correlation (*F_1,9_* = 57.8, *R^2^* = 0.87, *P* < 0.001). The regression was still significant when the outlying site (Hornussen) indicated in grey was included (*F_1,10_* = 23.1, *R^2^* = 0.70, *P* < 0.001). B ) The change in the SPEAR index downstream and the wastewater dilution factor calculated for average stream discharge (*Q_average_*) showed a significant relationship (*F_1,10_* = 11.1, *R^2^* = 0.53, *P* < 0.01). This was similar to the relationship between the SPEAR index change and the wastewater dilution factor calculated for the 95^th^ percentile of stream discharge (*Q_347_*) as shown in Fig.6D, Main text. In that regression, the outlying site of Hornussen was excluded for reasons explained in Appendix C.4.2.6. Shaded areas indicate 95% confidence intervals.

**
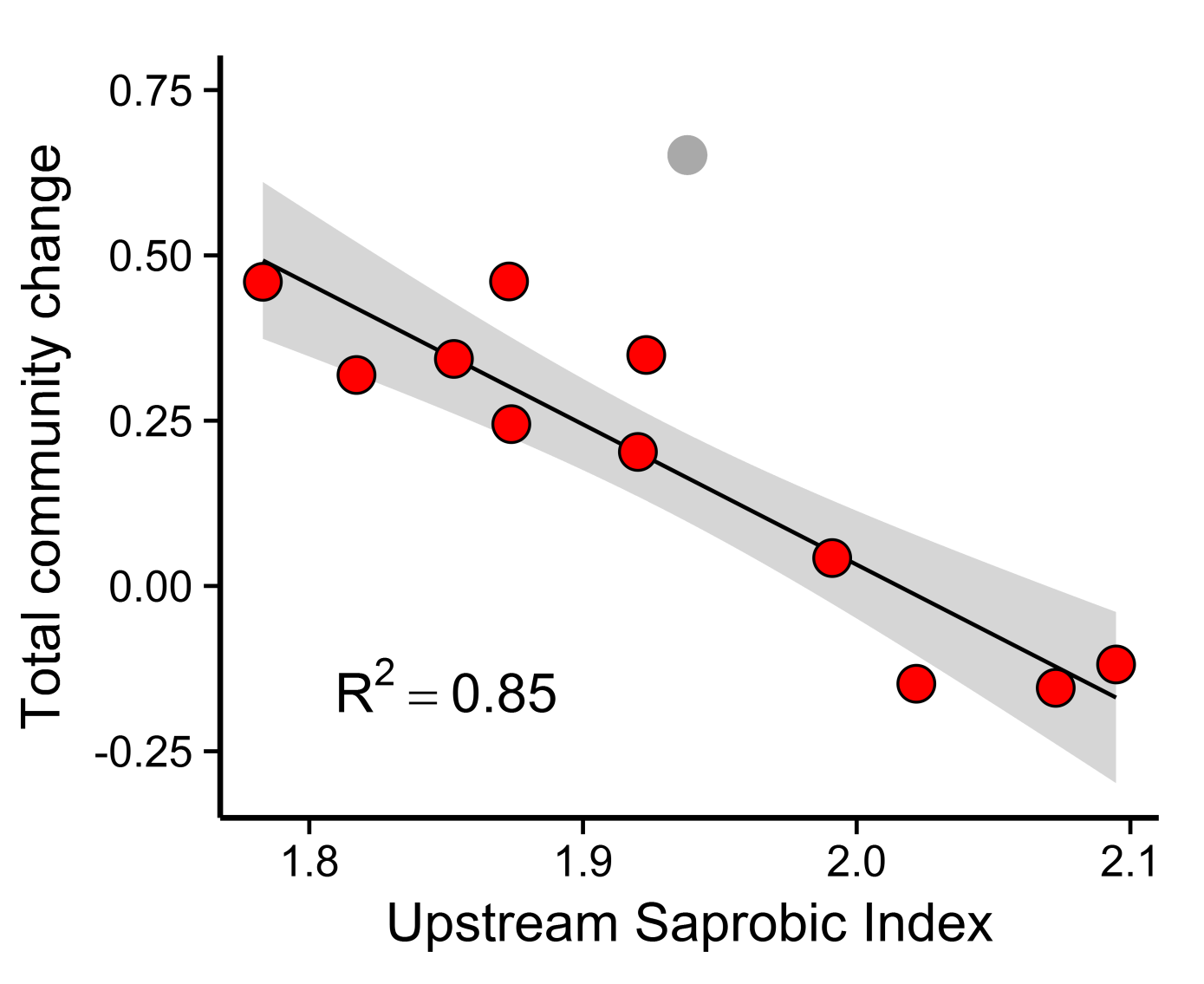
**

**Figure C6** The linear regression of total invertebrate community change and the mean Saprobic index (SI) scores for upstream sites showed a significant negative association (*F_1,9_* = 50.9, *R^2^* = 0.85, *P* < 0.001). This showed that communities in more degraded sites (i.e., those characterised by high SIs) were more resistant to wastewater perturbations. An outlying site (Niederdorf) is indicated in grey. The regression was still significant with the outlying site included (*F_1,10_* = 15.3, *R^2^* = 0.60, *P* < 0.01). Shaded areas indicate 95% confidence intervals.

**References**

Baselga, A. (2010) Partitioning the turnover and nestedness components of beta diversity. *Global Ecology and Biogeography,* **19,** 134-143.

Baselga, A. & Orme, C.D.L. (2012) betapart: an R package for the study of beta diversity. *Methods in Ecology and Evolution,* **3,** 808-812.

Beketov, M.A., Foit, K., Schäfer, R.B., Schriever, C.A., Sacchi, A., Capri, E., Biggs, J., Wells, C. & Liess, M. (2009) SPEAR indicates pesticide effects in streams – Comparative use of species- and family-level biomonitoring data. *Environmental Pollution,* **157,** 1841-1848.

Berger, W.H. & Parker, F.L. (1970) Diversity of planktonic Foraminifera in deep-sea sediments. *Science,* **168,** 1345-1347.

Borcard, D. & Legendre, P. (2002) All-scale spatial analysis of ecological data by means of principal coordinates of neighbour matrices. *Ecological Modelling,* **153,** 51-68.

Bunzel, K., Kattwinkel, M. & Liess, M. (2013) Effects of organic pollutants from wastewater treatment plants on aquatic invertebrate communities. *Water Research,* **47,** 597-606.

Burdon, F.J., McIntosh, A.R. & Harding, J.S. (2013) Habitat loss drives threshold response of benthic invertebrate communities to deposited sediment in agricultural streams. *Ecological Applications,* **23,** 1036-1047.

Clapcott, J.E., Young, R.G., Harding, J.S., Matthaei, C.D., Quinn, J.M. & Death, R.G. (2011) *Sediment Assessment Methods: protocols and guidelines for assessing the effects of deposited fine sediment on in-stream values*. Cawthron Institute, Nelson, New Zealand.

Cohen, J. (1992) A power primer. *Psychological Bulletin,* **112,** 155-159.

Fisher, R.A., Corbet, A.S. & Williams, C.B. (1943) The relation between the number of species and the number of individuals in a random sample of an animal population. *Journal of Animal Ecology,* **12,** 42–58.

Hurlbert, S.H. (1971) The non-concept of species diversity: a critique and alternative parameters. *Ecology,* **52,** 577-586.

Kempton, R.A. & Taylor, L.R. (1974) Log-series and log-normal parameters as diversity discriminators for Lepidoptera. *Journal of Animal Ecology,* **43,** 381–399.

Legendre, P. & Gallagher, E. (2001) Ecologically meaningful transformations for ordination of species data. *Oecologia,* **129,** 271-280.

Liechti, P. (2010) Methoden zur Untersuchung und Beurteilung der Fliessgewässer: Chemisch-physikalische Erhebungen, Nährstoffe. Umwelt-Vollzug 1005. BAFU, Bern. 44 pp.

Mac Nally, R. (2000) Regression and model-building in conservation biology, biogeography and ecology: the distinction between – and reconciliation of – ‘predictive’ and ‘explanatory’ models. *Biodiversity and Conservation,* **9,** 655–671.

Magurran, A.E. (2004) *Measuring Biological Diversity*. Blackwell Science Ltd, Cornwall, UK.

Oksanen, J., Blanchet, F.G., Kindt, R., Legendre, P., Minchin, P.R., O'Hara, R.B., Simpson, G.L., Solymos, P., Stevens, M.H.H. & Wagner, H. (2013) vegan: Community Ecology Package. R package version 2.0-7. <http://CRAN.R-project.org/package=vegan>.

Ortiz, J.D. & Puig, M.A. (2007) Point source effects on density, biomass and diversity of benthic macroinvertebrates in a Mediterranean stream. *River Research and Applications,* **23,** 155-170.

Peres-Neto, P.R., Jackson, D.A. & Somers, K.M. (2003) Giving meaningful interpretation to ordination axes: assessing loading significance in principal component analysis. *Ecology,* **84,** 2347-2363.

Pielou, E. (1975) *Ecological diversity*. Wiley, NJ, USA.

Quinn, G.P. & Keough, M.J. (2002) *Experimental Design and Data Analysis for Biologists*. Cambridge University Press, Cambridge, UK.

Quinn, J.M., Cooper, A.B., Davies‐Colley, R.J., Rutherford, J.C. & Williamson, R.B. (1997) Land use effects on habitat, water quality, periphyton, and benthic invertebrates in Waikato, New Zealand, hill‐country streams. *New Zealand Journal of Marine and Freshwater Research,* **31,** 579-597.

Schäfer, R.B., Caquet, T., Siimes, K., Mueller, R., Lagadic, L. & Liess, M. (2007) Effects of pesticides on community structure and ecosystem functions in agricultural streams of three biogeographical regions in Europe. *Science of The Total Environment,* **382,** 272-285.

Schaffner, M., Pfaundler, M. & Göggel, W. (2013) Fliessgewässertypisierung der Schweiz: Eine Grundlage für Gewässerbeurteilung und -entwicklung. Bundesamt für Umwelt, Bern. Umwelt-Wissen Nr. 1329: 63 S.

Schmidt-Kloiber, A. & Hering, D. (2015) [www.freshwaterecology.info](http://www.freshwaterecology.info) – An online tool that unifies, standardises and codifies more than 20,000 European freshwater organisms and their ecological preferences. *Ecological Indicators,* **53,** 271-282.

Schriever, C.A., Ball, M.H., Holmes, C., Maund, S. & Liess, M. (2007) Agricultural intensity and landscape structure: Influences on the macroinvertebrate assemblages of small streams in northern Germany. *Environmental Toxicology and Chemistry,* **26,** 346-357.

Shannon, C.E. (1948) A mathematical theory of communication. *The Bell System Technical Journal,* **27,** 379–423 and 623–656.

Stucki, P. (2010) Methoden zur Untersuchung und Beurteilung der Fliessgewasser: Makrozoobenthos Stufe F. Umwelt-Vollzug 1026. BAFU, Bern. 61 pp.

Szoecs, E. (2013) rSpear: Calculate SPEAR pesticides in R (<http://www.systemecology.eu/SPEAR/index.php)>. <http://cran.r-project.org/web/packages/rspear>.

Walsh, C. & Mac Nally, R. (2007) Hierarchical Partitioning Package. R Foundation for Statistical Computing, Vienna, Austria.
